# Factors That Promote H3 Chromatin Integrity during Transcription Prevent Promiscuous Deposition of CENP-A^Cnp1^ in Fission Yeast

**DOI:** 10.1371/journal.pgen.1002985

**Published:** 2012-09-20

**Authors:** Eun Shik Choi, Annelie Strålfors, Sandra Catania, Araceli G. Castillo, J. Peter Svensson, Alison L. Pidoux, Karl Ekwall, Robin C. Allshire

**Affiliations:** 1 Wellcome Trust Centre for Cell Biology, School of Biological Sciences, The University of Edinburgh, Edinburgh, Scotland, United Kingdom; 2 Department of Biosciences and Nutrition, Karolinska Institutet, NOVUM, Huddinge, Sweden; Duke University, United States of America

## Abstract

Specialized chromatin containing CENP-A nucleosomes instead of H3 nucleosomes is found at all centromeres. However, the mechanisms that specify the locations at which CENP-A chromatin is assembled remain elusive in organisms with regional, epigenetically regulated centromeres. It is known that normal centromeric DNA is transcribed in several systems including the fission yeast, *Schizosaccharomyces pombe*. Here, we show that factors which preserve stable histone H3 chromatin during transcription also play a role in preventing promiscuous CENP-A^Cnp1^ deposition in fission yeast. Mutations in the histone chaperone FACT impair the maintenance of H3 chromatin on transcribed regions and promote widespread CENP-A^Cnp1^ incorporation at non-centromeric sites. FACT has little or no effect on CENP-A^Cnp1^ assembly at endogenous centromeres where CENP-A^Cnp1^ is normally assembled. In contrast, Clr6 complex II (Clr6-CII; equivalent to Rpd3S) histone deacetylase function has a more subtle impact on the stability of transcribed H3 chromatin and acts to prevent the ectopic accumulation of CENP-A^Cnp1^ at specific loci, including subtelomeric regions, where CENP-A^Cnp1^ is preferentially assembled. Moreover, defective Clr6-CII function allows the *de novo* assembly of CENP-A^Cnp1^ chromatin on centromeric DNA, bypassing the normal requirement for heterochromatin. Thus, our analyses show that alterations in the process of chromatin assembly during transcription can destabilize H3 nucleosomes and thereby allow CENP-A^Cnp1^ to assemble in its place. We propose that normal centromeres provide a specific chromatin context that limits reassembly of H3 chromatin during transcription and thereby promotes the establishment of CENP-A^Cnp1^ chromatin and associated kinetochores. These findings have important implications for genetic and epigenetic processes involved in centromere specification.

## Author Summary

Centromeres are the chromosomal locations at which kinetochores, the machinery that directs chromosome segregation, are assembled. In most eukaryotes, centromere location is epigenetically determined, meaning that the underlying DNA sequence does not dictate where they are formed. The genome is packaged in particles called nucleosomes, composed of histone proteins. Centromere DNA is wrapped around unusual nucleosomes that differ from those elsewhere in the genome because the histone H3-like CENP-A replaces the normal histone H3 component. We used fission yeast to investigate where CENP-A^Cnp1^ nucleosomes are formed in cells containing excess CENP-A^Cnp1^ and how the formation of these non-centromeric CENP-A nucleosomes is controlled. H3 nucleosomes are disassembled and reassembled during transcription by RNA polymerase II (RNAPII). We show that the normal process of reassembling robust H3 chromatin on RNAPII genes is required to prevent CENP-A^Cnp1^ assembly in its place. Centromeres allow a low level of RNAPII transcription, and our analyses suggest that DNA sequences and chromatin contexts at centromeres may limit the activities required to stabilize and reassemble H3 chromatin during transcription in order to promote the establishment of CENP-A^Cnp1^ chromatin and associated kinetochores.

## Introduction

Centromere formation is influenced by both genetic and epigenetic processes (reviewed in [1], [2], [3], [4], [5], [6]). The fundamental feature that defines active centromeres resides at the chromatin level; the presence of specialized chromatin in which histone H3 is replaced by the conserved H3 variant CENP-A (CenH3). CENP-A is highly enriched at active centromeres and is indispensible for centromere function. CENP-A chromatin provides a platform for recruiting kinetochore proteins which in turn direct CENP-A^Cnp1^ targeting and retention; thus CENP-A chromatin serves as an epigenetic mark allowing propagation of centromeres at specific loci [1], [5], [7].

Fission yeast (*Schizosaccharomyces pombe*) centromeres provide an excellent model for dissecting the mechanism of CENP-A chromatin assembly [8]. CENP-A^Cnp1^ chromatin assembles on 7-10 kb central domain regions which, as at mammalian centromeres, are surrounded by heterochromatin formed by methylation of histone H3 lysine 9 [5]. We previously showed that flanking heterochromatin is required to establish CENP-A^Cnp1^ on the central domain of naïve plasmid-based minichromosome DNA [9], [10]. However, once established, CENP-A^Cnp1^ chromatin is propagated in the absence of the adjacent heterochromatin. The epigenetic nature of CENP-A^Cnp1^ assembly in fission yeast is underscored by the findings that neocentromeres can form at subtelomeric regions and centromeres can be inactivated on dicentric chromosomes [11], [12]. The assembly of CENP-A^Cnp1^ chromatin at novel secondary sites could be beneficial for the rescue of acentric chromosomes but detrimental if activated on normal chromosomes. Dicentric chromosomes are highly unstable and thus mechanisms must operate to suppress assembly of CENP-A^Cnp1^ chromatin at such secondary sites (reviewed in [6]). Related to this, CENP-A is overexpressed and more CENP-A is incorporated at centromeres in some tumor cells [13], . CENP-A overexpression can trigger neocentromere formation resulting in dicentric chromosomes and consequential genome instability that drives tumor progression [15], [16], [17]. Thus mechanisms that prevent promiscuous deposition of CENP-A into non-centromeric chromatin are important for preventing genome instability and provide insight into the processes that normally allow CENP-A deposition.

Accumulating evidence in several organisms indicates that transcription occurs at centromeres and neocentromeres [18], [19], [20], [21], [22], [23], however it is unclear how transcription might influence CENP-A deposition mechanistically. Recently, we demonstrated that non-coding RNAs are transcribed by RNA polymerase II from the central CENP-A^Cnp1^ chromatin domains of fission yeast centromeres [24]. It is well known that advancing RNAPII promotes the disassembly of nucleosomes in its path and this mediates the eviction of H3.1 and its replacement with H3.3 in metazoa (reviewed in [25], [26]). Similarly, transcription-coupled nucleosome disassembly may allow the exchange of H3 for CENP-A within centromeres. At promoters, chromatin remodeling and histone acetylation destabilize or remove nucleosomes to allow transcription factors and RNAPII access to the DNA template. During transcriptional elongation, nucleosomes in front of RNAPII are transiently disassembled and histone chaperones, such as FACT (Facilitates chromatin transcription) and Spt6, act to recycle the dissociated histones so that the same histones are reassembled in nucleosomes behind elongating RNAPII [27], [28], [29]. Moreover, acetylation of nucleosomes ahead of RNAPII appears to facilitate the passage of RNAPII through chromatin, but this acetylation must be removed to restore chromatin to its original stable state within coding regions. In *Saccharomyces cerevisiae*, the Rpd3S histone deacetylase (HDAC) complex deacetylates histones in coding regions [30], [31]. The integrity of transcribed chromatin has been shown to be weakened in cells with defective FACT, Spt6 or Rpd3S and as a consequence cryptic transcription initiates from within coding regions to produce spurious intragenic transcripts [28], [31], [32]. *S. pombe* Clr6 Complex II (Clr6-CII) performs the same function as *S. cerevisiae* Rpd3S and is composed of related subunits (Clr6, Pst2, Cph1, Cph2, Alp13, Prw1; Clr6 = Rpd3) [33].

Here we investigate the contribution of factors that govern nucleosome dynamics during RNAPII transcription to CENP-A^Cnp1^ deposition. We find that defects in FACT and Clr6-CII, which normally restore H3 chromatin integrity during RNAPII transcription, cause increased CENP-A^Cnp1^ incorporation at non-centromeric loci when CENP-A^Cnp1^ levels are elevated. FACT mutants show a strong phenotype, severely disrupt H3 chromatin integrity on RNAPII genes and promote widespread mis-incorporation of CENP-A^Cnp1^. In contrast, cells with defective Clr6-CII display a more subtle disturbance of H3 chromatin integrity and allow increased CENP-A^Cnp1^ incorporation at more specific locations, including subtelomeric regions. Remarkably, Clr6-CII mutants allow the *de novo* assembly of CENP-A^Cnp1^ and kinetochore proteins on plasmids carrying centromeric central domain DNA alone and thus circumvent the requirement for flanking heterochromatin in the establishment of CENP-A^Cnp1^ chromatin. We propose that CENP-A^Cnp1^ chromatin can assemble *de novo* on particular genomic regions when the integrity of H3 chromatin fails to be restored during transcription-induced chromatin reconfiguration. By extrapolation, at endogenous centromeres, specific chromatin contexts such as flanking heterochromatin (provided by repetitive elements) or pre-established centromeric chromatin may alter nucleosome dynamics during RNAPII transcription to facilitate the replacement of H3 by CENP-A^Cnp1^.

## Results

### FACT mutants are hypersensitive to CENP-A^Cnp1^ overexpression

**Figure 1:**
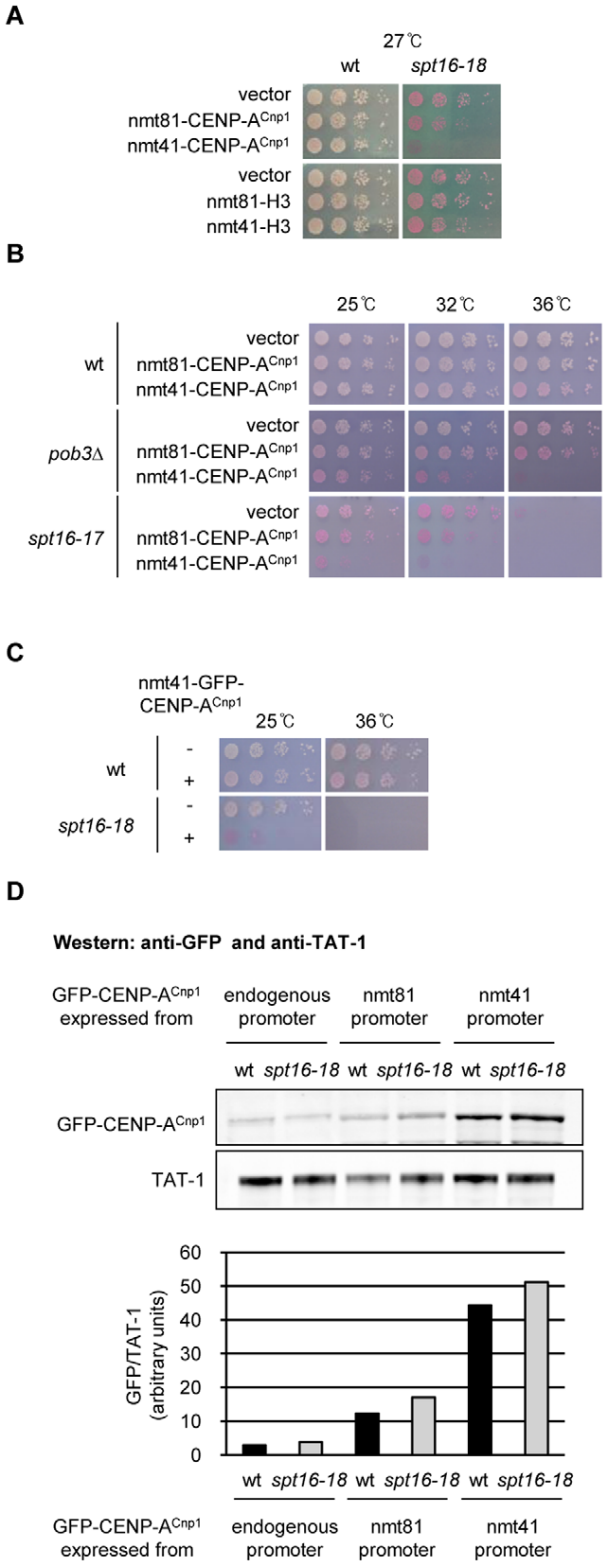
Overexpression of CENP-A^Cnp1^ causes toxicity in FACT mutants. (A) Viability of wild-type (wt) and *spt16-18* cells expressing additional CENP-A^Cnp1^ or H3 at low (nmt81-CENP-A^Cnp1^, nmt81-H3) or medium (nmt41-CENP-A^Cnp1^, nmt41-H3) levels compared to empty vector. Cells were grown at 27°C which is semi-permissive for *spt16-18*. Phloxine B plates stain dead cells red. (B) Viability of wt, *pob3*Δ and *spt16-17* cells expressing additional CENP-A^Cnp1^ at low (nmt81-CENP-A^Cnp1^) or medium (nmt41-CENP-A^Cnp1^) levels compared to empty vector at indicated temperatures. (C) Viability of wt and *spt16-18* cells expressing GFP-CENP-A^Cnp1^ from integrated pREP41-GFP-*cnp1* ^+^ (nmt41-GFP-CENP-A^cnp1^) compared to no GFP-CENP-A^Cnp1^ control. (D) Western analysis of GFP-CENP-A^Cnp1^ levels in wt and *spt16-18* cells expressing GFP-CENP-A^Cnp1^ under endogenous, nmt81 or nmt41 promoter (upper panel). The intensities of GFP-CENP-A^Cnp1^ and TAT-1 (alpha-tubulin) signals were measured using LICOR Odyssey Infrared Imaging System software (Li-COR Bioscience) and the relative intensities of GFP-CENP-A^Cnp1^/TAT-1 were quantified (bottom panel). GFP-CENP-A^Cnp1^ was expressed for 24 h at 25°C before harvest.

FACT is composed of two evolutionarily conserved subunits, Spt16 and Pob3 (SSRP1 in human) [34], [35]. In budding yeast both FACT subunits are essential, but fission yeast requires only Spt16 for survival [36]. Thus, Spt16 performs the major functions of FACT in fission yeast. To fully assess whether FACT modulates CENP-A^Cnp1^ deposition in fission yeast, we generated temperature sensitive (*ts*) alleles of *spt16* ^+^. Cells with a defect in mechanisms that prevent inappropriate CENP-A^Cnp1^ deposition in non-centromeric chromatin are expected to be sensitive to CENP-A^Cnp1^ overexpression. We noticed that elevated CENP-A^Cnp1^ levels (OE-CENP-A^Cnp1^) exacerbate the *ts* phenotypes in *spt16-18* cells (Figure 1A; for generation of *spt16-ts* alleles see Figure S1A). Since different *spt16* alleles and *pob3*Δ also exhibit sensitivity, this phenotype is not allele or subunit specific (Figure 1B and 1C). The effect was greater when CENP-A^Cnp1^ was expressed from the stronger promoter nmt41 (nmt promoters are described in [37]). The nmt41 promoter produces 4-fold more GFP-CENP-A^Cnp1^ than the weaker nmt81 version (Figure 1D). The level of OE-CENP-A^Cnp1^ expressed from *nmt41-GFP-cnp1* ^+^ and *nmt81-GFP-cnp1* ^+^ is comparable in wild-type (wt) and *spt16-18* cells (Figure 1D). This interaction is specific to CENP-A^Cnp1^ since *spt16-18* cells are not sensitive to elevated expression of histone H3 (Figure 1A; see [38]).

Marker genes placed in the central domain of fission yeast centromeres are normally silenced and mutations affecting CENP-A^Cnp1^ deposition exhibit decreased silencing [39]. Silencing of *ura4* ^+^ in the central domain (*cnt1*:*ura4* ^+^) is largely unaffected in *pob3*Δ cells and in *spt16-ts* alleles at the permissive temperature (25°C) (Figure S1B and S1C). Factors involved in CENP-A^Cnp1^ deposition at centromeres often show genetic interactions when combined with defects in CENP-A^Cnp1^ itself [39]. However, *pob3*Δ does not display reduced growth when combined with *cnp1-87* which has a relatively weak mutation in CENP-A^Cnp1^ (Figure S1D). One explanation for the sensitivity of FACT mutants to elevated CENP-A^Cnp1^ levels but the lack of an interaction with CENP-A^Cnp1^ mutants is that FACT is required to prevent the promiscuous incorporation of CENP-A^Cnp1^ in place of H3 at non-centromeric locations and is not directly involved in maintaining CENP-A^Cnp1^ chromatin at centromeres. The mis-incorporation of CENP-A^Cnp1^ at other locations in FACT mutants may cause cell lethality by interfering with normal chromatin-based processes such as transcription or possibly due to the induction of ectopic kinetochores.

### Spt16 is required to maintain the integrity of H3 chromatin on RNAPII genes

It is well known that FACT is required for the RNAPII transcription-coupled reassembly of chromatin on transcription units in *S. cerevisiae* [40], [41]. However, although the reassembly of chromatin on transcribed templates with recycled histones is defective in *spt16* mutants, the assembly of chromatin from the free histone pool remains active so that new histones can be incorporated. Indeed, the elevated loss of nucleosomes from transcription units in *spt16* mutants enhances their replenishment with new histones from the free pool [40]. Thus FACT normally prevents the incorporation of free histones by recycling pre-existing histones in template-associated nucleosomes during transcription. Our previous analyses indicate that CENP-A^Cnp1^ and H3 can compete for incorporation into chromatin at centromeres [38]. If FACT mutants cause elevated turnover of H3 nucleosomes on RNAPII-transcribed templates in *S. pombe* then this might provide the opportunity for CENP-A^Cnp1^ to replace H3 in non-centromeric transcribed chromatin.

**Figure 2:**
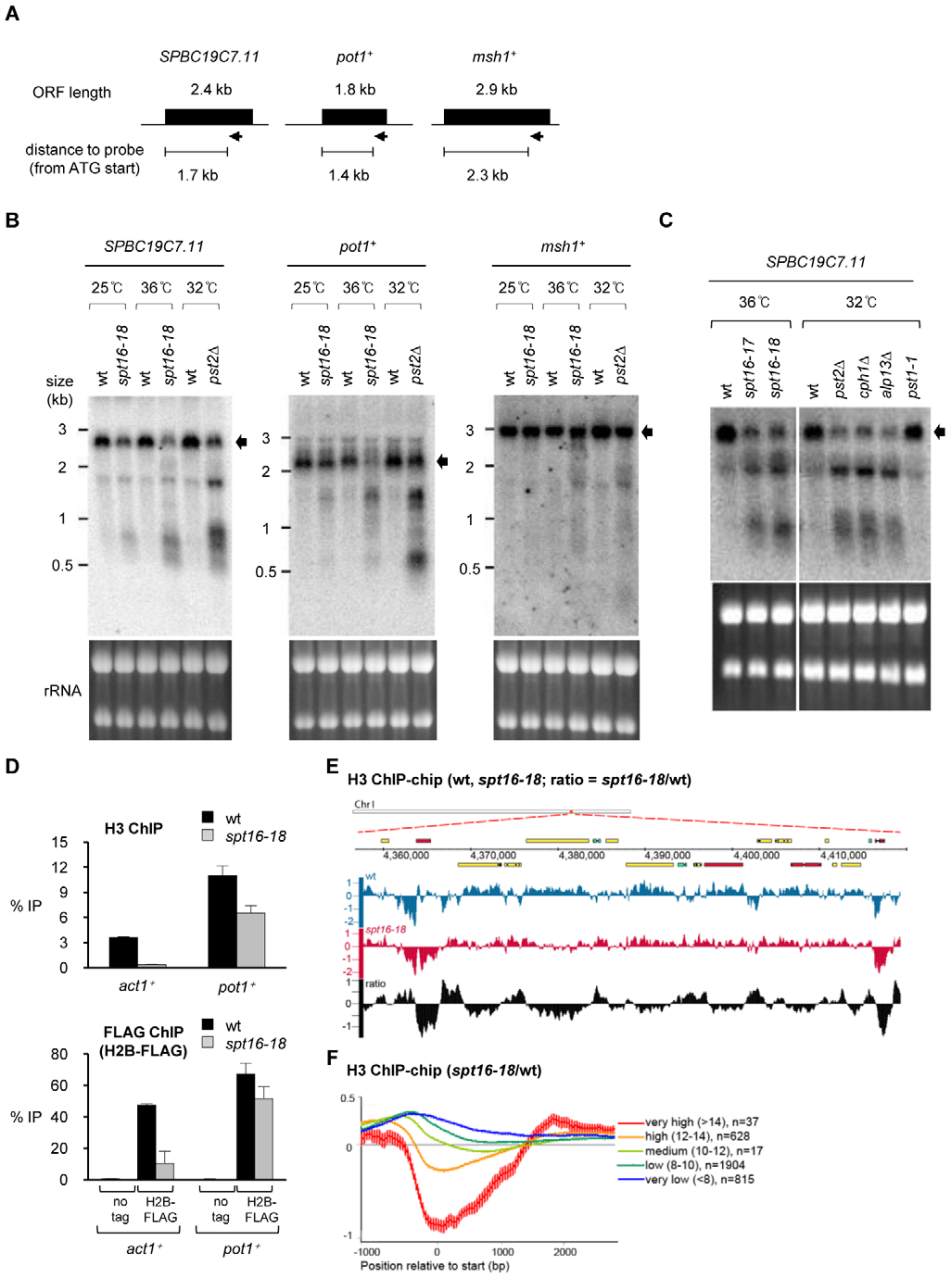
Spt16 is required to suppress cryptic transcription initiation and nucleosome loss at RNAPII genes. (A) Schematic of genes and positions of RNA probes (arrow) used in Northern analysis. (B) Northern analyses of transcripts from *SPBC19C7.11*, *pot1* ^+^ and *msh1* ^+^ genes. RNA was extracted from cells grown at 25°C (wt, *spt16-18*), 32°C for 6 h (wt, *pst2*Δ) or 36°C for 1 h (wt, *spt16-18*) after shift from 25°C. Arrow indicates full-length transcripts. (C) Northern analysis of transcripts from *SPBC19C7.11*. Cells were grown at 36°C for 2 h after shift from 25°C (wt, *spt16-ts*) or at 32°C for 6 h after shift from 25°C (wt, *pst2*Δ, *cph1*Δ, *alp13*Δ, *pst1-1*), as indicated. (D) ChIP analysis of H3 levels at *act1* ^+^ and *pot1* ^+^ in wt and *spt16-18* cells grown at 36°C for 1 h after shift from 25°C (top). ChIP analysis of H2B levels at *act1* ^+^ and *pot1* ^+^ in wt and *spt16-18* cells expressing H2B-FLAG or untagged H2B (bottom). Enrichment is reported as % IP. Error bar indicates S.D. from 3 biological replicates. (E) Genome browser view showing ChIP-chip occupancy profiles for H3 in wt (blue) and *spt16-18* cells (red). The relative ratio (*spt16-18*/wt) is indicated in black. Data on the Y-axis are presented in log2 scale and the X-axis shows genome positions in base pairs. Open reading frames (ORFs) are displayed as boxes and colored according to transcription levels (highly transcribed genes in red, medium transcribed genes in green and low transcribed genes in blue). (F) Average gene analysis for the ratio of H3 occupancy in *spt16-18* mutants versus wt. Genes are aligned at transcription start site and divided into four groups dependent of their transcription levels. Data on the Y-axis are presented in log2 scale and the X-axis shows position relative to start (bp). Values for gene expression were calculated using Podbat based on the RNA data from a previous study [61], [62]. The gene expression value ranged between 5 and 15, and genes were assigned into categories based on this value. Five categories were made: very high (>14) (n = 37), high (12–14) (n = 591), medium (10–12) (n = 1726), low (8–10) (n = 1904) and very low (<8) (n = 815). n = number of genes in each group. Error bars represent 99% confidence intervals.

We first tested if, as in *S. cerevisiae*, FACT is also required for the reassembly of chromatin on genes transcribed by RNAPII in *S. pombe*. The detection of shorter sense and antisense transcripts initiated from within open reading frames (ORFs) is a hallmark of defective RNAPII transcription-coupled chromatin reassembly [28], [31], [32]. Clr6-CII is the *S. pombe* equivalent of Rpd3S in *S. cerevisiae* and microarray expression profiling previously indicated the presence of antisense transcripts from a set of genes in *S. pombe* cells with defective Clr6-CII [33]. We reasoned that the same genes may display a similar defect in FACT mutants and selected three genes (*SPBC197.11*, *pot1^+^* and *msh1^+^*), for northern analyses in wild-type (wt), *spt16-18* and *pst2*Δ (Pst2; Sin3-related Clr6-CII subunit) cells (Figure 2A). Probes complementary to 3′ region of these genes detect short, abnormal transcripts in cells with defective Spt16 or Pst2 (Figure 2B and 2C) and the transcript sizes suggest that they originate from within the ORFs. Clr6-CII preferentially targets transcribed regions to suppress aberrant transcription initiation, whereas Clr6 complex I (Clr6-CI) acts at promoters [33]. Consistent with this, spurious intragenic transcripts are detected in cells lacking other Clr6-CII subunits (*cph1*Δ, *alp13*Δ) but not in mutants that specifically affect Clr6-CI (*pst1-1*; Figure 2C).

To directly determine if chromatin integrity is compromised in *S. pombe* cells with defective FACT we measured the levels of H3 and H2B on the *pot1* ^+^ gene where aberrant intragenic transcripts were readily detected in *spt16-18* cells. We also analyzed the *act1* ^+^ gene, which is highly transcribed and thus associated nucleosomes are likely to be more dynamic. ChIP analyses indicated that the levels of histones are dramatically reduced on *act1* ^+^ in *spt16-18* cells compared to wild-type cells (Figure 2D). A modest reduction of histone levels is also observed on *pot1* ^+^ in *spt16-18* cells. This suggests that Spt16 is required to maintain H3 nucleosomes on *act1* ^+^ and *pot1* ^+^. To further assess loss of chromatin integrity from RNAPII genes in *spt16-18* cells, we performed genome-wide analyses of H3 association in wt and *spt16-18* cells. We observed a widespread decrease in the relative levels of H3 on RNAPII genes in *spt16-18* cells (Figure 2E). The reduction of H3 levels on RNAPII genes in *spt16-18* cells is correlated with their level of transcription (Figure 2F). Thus, as in *S. cerevisiae*, FACT is required to maintain canonical H3 nucleosomes on genes during transcription by RNAPII.

### CENP-A^Cnp1^ accumulates at non-centromeric locations in cells with defective FACT

**Figure 3:**
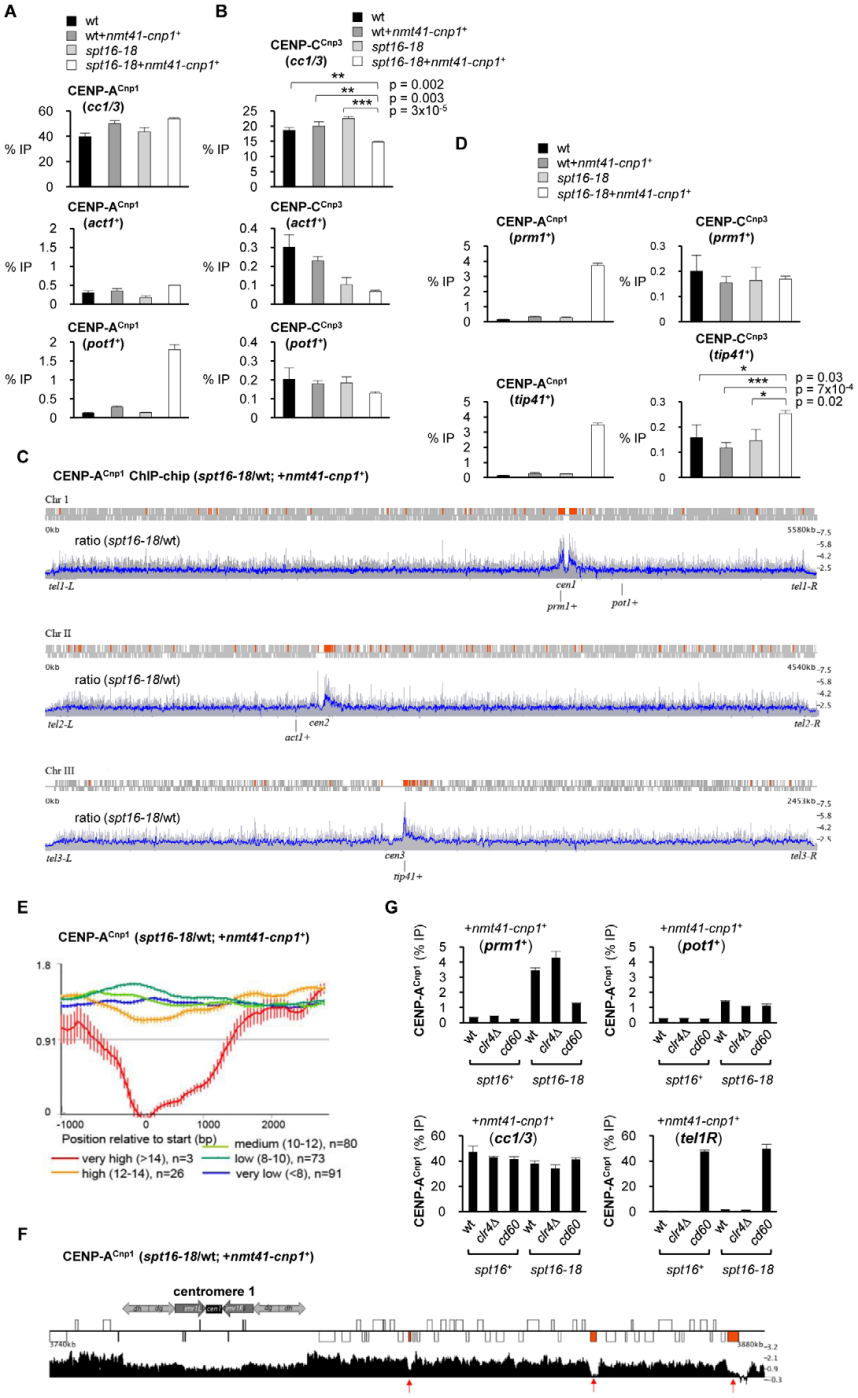
Spt16 prevents promiscuous incorporation of CENP-A^Cnp1^. (A) ChIP analysis of CENP-A^Cnp1^ levels at endogenous centromeres (*cc1/3*), *act1* ^+^ and *pot1* ^+^ in wt and *spt16-18* cells in the absence or presence of OE-CENP-A^Cnp1^ (*nmt41-cnp1* ^+^; pREP41-*cnp1* ^+^ integrated at *ars1* locus). Cells were grown at 36°C for 1 h after shift from 25°C. (B) ChIP analysis of CENP-C^Cnp3^ in the same samples. (C) ChIP-chip: relative CENP-A^Cnp1^ levels in *spt16-18* cells compared to wt. ORFs are displayed as grey boxes. Regions of at least 1 kb in length and with >2-fold increase in CENP-A^Cnp1^ signal above genome-wide average are colored red. Data on the Y-axis are presented in linear scale. Blue: running average signal/100 probes. Grey: signal for individual probes. (D) ChIP analyses of CENP-A^Cnp1^ and CENP-C^Cnp3^ levels at *prm1* ^+^ and *tip41* ^+^. Error bars indicate S.D. from 3 biological replicates. (E) Average gene analysis for the ratio of CENP-A^Cnp1^ occupancy in *spt16-18* versus wt cells (with OE-CENP-A^Cnp1^). Genes within 100 kb of centromeres were selected and divided into four groups dependent of their relative transcription levels and aligned at their transcriptional start sites. Data on the Y-axis are presented in log2 scale and the X-axis shows position relative to start (bp). Values for gene expression were calculated using Podbat based on the RNA data from a previous study [61], [62]. The gene expression value ranged between 5 and 15, and genes were assigned into categories based on this value. Five categories were made: very high (>14) (n = 37), high (12–14) (n = 591), medium (10–12) (n = 1726), low (8–10)(n = 1904) and very low (<8) (n = 815). n = number of genes in each group. Error bars represent 99% confidence intervals. (F) Genome browser view showing relative enrichment of CENP-A^Cnp1^ at *cen1*-proximal regions (*spt16-18*/wt; with OE-CENP-A^Cnp1^). Data on the Y-axis are presented in linear scale. Boxes are ORFs; red: genes with very high expression levels. (G) ChIP analysis of CENP-A^Cnp1^ levels at *prm1* ^+^, *pot1* ^+^, endogenous centromeres (*cc1/3*) and a neocentromere region (*tel1R*) in wt, *clr4*Δ and *cd60* (neocentromere strain; *cen1* DNA deleted) cells containing either *spt16* ^+^ or *spt16-18* allele and all with OE-CENP-A^Cnp1^ (*nmt41-cnp1* ^+^). Note: centromere primers (*cc1/3*) detect both *cen1* and *cen3* and thus CENP-A^Cnp1^ enrichment at *cc1/3* in *cd60* represents CENP-A^Cnp1^ levels at *cen3* only. In all ChIP analyses, enrichment is reported as % IP.

Normally, cells express low levels of CENP-A and this together with robust and specific mechanisms involving centromere associated assembly factors maintains CENP-A exclusively at centromeres. In order to determine if defective FACT function allows the incorporation of CENP-A^Cnp1^ at non-centromeric locations, we compared the levels of CENP-A^Cnp1^ on *act1* ^+^ and *pot1* ^+^ genes in wild-type and *spt16-18* cells expressing additional CENP-A^Cnp1^. CENP-A^Cnp1^ incorporation into the chromatin covering *pot1* ^+^, but not *act1*, was substantially increased in *spt16-18* relative to wild-type cells expressing excess CENP-A^Cnp1^ (*nmt41-cnp1* ^+^; Figure 3A). The *spt16-18* mutation alone or OE-CENP-A^Cnp1^ in wild-type cells does not significantly affect the levels of CENP-A^Cnp1^ on *pot1* ^+^. The increased accumulation of CENP-A^Cnp1^ on *pot1* ^+^ compared to *act1* ^+^ in *spt16-18* cells is intriguing since H3 occupancy is more severely reduced on *act1* ^+^ (Figure 2D). The *act1* ^+^ gene is very highly expressed and even in wild-type cells it retains a relatively low level of H3. We surmise that, like H3, CENP-A^Cnp1^ cannot stably assemble on highly transcribed genes such as *act1* ^+^ in *spt16-18* cells due to its continual removal. But on genes such as *pot1* ^+^ that are transcribed at intermediate levels, H3 or CENP-A^Cnp1^ can be stably incorporated from the free histone pool.

It is well established that CENP-A chromatin serves as a platform for assembly of kinetochore proteins at centromeres [42]. The expression of additional CENP-A^Cnp1^ in FACT defective *spt16-18* cells results in a modest but reproducible reduction in the association of the CENP-C^Cnp3^ kinetochore protein with centromeres (Figure 3B). One explanation is that the pool of endogenous CENP-C^Cnp3^ is limited and the promiscuous incorporation of CENP-A^Cnp1^ at many non-centromeric locations leads to the redistribution of this CENP-C^Cnp3^. However, CENP-C^Cnp3^ levels were not observed to increase on *act1* ^+^ or *pot1* ^+^ in *spt16-18* expressing additional CENP-A^Cnp1^ (*nmt41-cnp1* ^+^; Figure 3B). Other regions in the genome may accumulate higher levels of CENP-A^Cnp1^ allowing them to attract CENP-C^Cnp3^ away from centromeres. To explore this possibility, we compared the genome-wide distribution of overexpressed CENP-A^Cnp1^ in wild-type and *spt16-18* cells. The absolute levels of CENP-A^Cnp1^ association were quantified across the genome using ChIP-chip. In *spt16-18* cells expressing excess CENP-A^Cnp1^ (*nmt41-cnp1* ^+^), a 1.7 fold global increase in chromosomal levels of CENP-A^Cnp1^ occurs. The most notable accumulation relative to wild-type cells is observed on the euchromatic regions adjacent to centromeric heterochromatin (Figure 3C and Figure S2; also see Table S1 for CENP-A^Cnp1^ and H3 enrichment at selected genes). To further evaluate this, we conducted a genome-wide data-driven search for regions of CENP-A^Cnp1^-chromatin association. Regions of at least 1 kb in length and with more than 2-fold increase in CENP-A^Cnp1^ signal above genome-wide average were selected. 168 regions were identified, and 96 of these were within 100 kb of centromeres (Red boxes; Figure 3C), i.e. 57% of regions found in 4.8% of the genome. This was found to be highly significant (p<10^-84^, hypergeometric distribution). ChIP-qPCR analyses confirm that high levels of CENP-A^Cnp1^ accumulate on *prm1* ^+^ (high expression class) and *tip41* ^+^ (very low expression class) which are proximal to *cen1* and *cen3*, respectively (Figure 3D). Increased association of CENP-C^Cnp3^ is detected on *tip41* ^+^ in *spt16-18* OE-CENP-A^Cnp1^ cells, indicating that other kinetochore proteins can be recruited to some non-centromeric sites (Figure 3D). Only genes with internal cryptic transcriptional start sites will generate short aberrant intragenic transcripts. Indeed, short cryptic transcripts are not readily detected from *prm1* ^+^ and *tip41* ^+^ in *spt16-18* cells and *pst2*Δ cells (Figure S3A). Also, short cryptic transcripts were not evident from *act1* ^+^ (Figure S3B). Thus, it is unlikely that cryptic transcription itself is the primary cause of H3 loss or CENP-A^Cnp1^ incorporation in FACT mutants. ChIP-chip analyses show that the levels of H3 and CENP-A^Cnp1^ are lowest on transcribed genes with high levels of expression and this decreases further in *spt16-18* cells compared to wild-type cells (Figure S4). Even within centromere proximal regions where overexpressed CENP-A^Cnp1^ is preferentially assembled in *spt16-18* cells, very highly transcribed genes do not allow CENP-A^Cnp1^ accumulation (Figure 3E and 3F). This supports the conclusion that genes with low to intermediate levels of RNAPII transcription permit more stable incorporation of CENP-A^Cnp1^ in the absence of FACT function.

### Centromere activity is required for the incorporation of excess CENP-A^Cnp1^ in centromere proximal regions

The preferential accumulation of CENP-A^Cnp1^ close to centromeres in FACT defective *spt16-18* cells overexpressing CENP-A^Cnp1^ may be dependent on centromeric heterochromatin or on the presence of an active centromere. Centromeric heterochromatin is completely dependent on *clr4* ^+^, encoding the only H3K9 methyltransferase in *S. pombe*, which is non-essential. We have previously shown that heterochromatin is required for the *de novo* assembly of CENP-A^Cnp1^ on nearby centromeric DNA. However, increased CENP-A^Cnp1^ association was still detected on *prm1* ^+^ in *spt16-18* cells overexpressing CENP-A^Cnp1^ that lack Clr4 (*clr4*Δ; Figure 3G). Therefore to determine if the accumulation of CENP-A^Cnp1^ is directed by the presence of an active centromere, we used *cd60* cells in which the normal *cen1* was deleted and a neocentromere formed in the *tel1R* subtelomeric region [12]. In these cells, enrichment of CENP-A^Cnp1^ on *prm1* ^+^ (∼20 kb away from the *cen1* deletion) is significantly reduced in *spt16-18* cells overexpressing CENP-A^Cnp1^ but the association of CENP-A^Cnp1^ with the non-centromeric *pot1* ^+^ gene is unaffected (Figure 3G). The 40 kb deletion in *cd60* cells, completely removes both the central CENP-A^Cnp1^/kinetochore and heterochromatin domains from *cen1*. This indicates that it is the pre-assembled centromere-kinetochore, rather than heterochromatin, that promotes the assembly of CENP-A^Cnp1^ over adjacent euchromatin in FACT defective *spt16-18* cells. Pre-assembled centromeres must attract excess CENP-A^Cnp1^ and deposition factors which aid the assembly of CENP-A^Cnp1^ chromatin over nearby euchromatin when it becomes permissive for CENP-A^Cnp1^ incorporation in cells with compromised FACT function.

### Cells with defective Spt6 are sensitive to CENP-A^Cnp1^ overexpression

If RNAPII transcription-coupled nucleosome reassembly acts to exclude CENP-A^Cnp1^ from incorporation at non-centromeric regions, then other mutants affecting this process should also allow mis-incorporation of CENP-A^Cnp1^. In *S. cerevisiae*, the Spt6 chaperone also acts to reassemble nucleosomes within ORFs following transcription [28]. Our analyses demonstrate that *S. pombe* cells bearing the *spt6-1* mutation [43] are also sensitive to elevated CENP-A^Cnp1^ levels (Figure S5A). In addition, *spt6-1* also allows this CENP-A^Cnp1^ to accumulate at non-centromeric regions (Figure S5B). Thus both the FACT and Spt6 chaperones, which are required to maintain chromatin integrity on genes following transcription by RNAPII, are implicated in preventing the misincorporation of CENP-A^Cnp1^ when it is expressed at elevated levels.

### FACT is required for the preferential incorporation of CENP-A^Cnp1^ at specific locations

Neocentromeres can form at subtelomeric regions in *S. pombe* following removal of the normal centromere [12]. The expression of additional CENP-A^Cnp1^ in wild-type cells also allows its accumulation over these telomere adjacent regions (Castillo et al in preparation; see Figure S6A). This suggests that subtelomeric regions possess particular features that make them favorable substrates for CENP-A^Cnp1^ assembly. Interestingly, our ChIP-chip analyses reveal that CENP-A^Cnp1^ does not accumulate over subtelomeric regions in FACT defective *spt16-18* cells expressing additional CENP-A^Cnp1^, even though they are normally preferred sites for the accumulation of additional CENP-A^Cnp1^ in wild-type cells (Figure 3C - subtelomeric CENP-A^Cnp1^ accumulation does not increase in *spt16-18* relative to wild-type cells). This suggests that FACT is not normally active, or is unable to prevent CENP-A^Cnp1^ deposition, at wild-type subtelomeric regions.

To explore the relationship between FACT and CENP-A^Cnp1^ permissive regions further we examined the effect of defective FACT function on the association of CENP-A^Cnp1^ with centromeric DNA in both its normal context and at an ectopic location. CENP-A^Cnp1^ normally assembles on the central domain regions of centromeres due to kinetochore-mediated CENP-A^Cnp1^ recruitment and/or maintenance mechanisms. Centromeric heterochromatin also aids the assembly of CENP-A^Cnp1^ chromatin at centromeres [9], [10]. However, in addition to these extrinsic influences, central domain DNA itself may possess intrinsic sequence-driven features that promote CENP-A^Cnp1^ assembly. To examine this, we constructed a strain in which the entire 8.6 kb from the central domain of *cen2* was inserted at the euchromatic *ura4* ^+^ locus (*ura4* ^+^ *-int-cc2*). This separates central domain DNA on which CENP-A^Cnp1^ chromatin normally assembles from flanking heterochromatin (Figure S6B). In cells expressing additional CENP-A^Cnp1^ we detected substantially greater levels of CENP-A^Cnp1^ over *ura4* ^+^ *-int-cc2* than on the non-centromeric *act1* ^+^ or *pot1* ^+^ loci (Figure S6C). This indicates that even when central domain DNA is placed outside the context of a normal centromere it has an innate ability to attract CENP-A^Cnp1^. We conclude that *cc2* central domain DNA must possess particular features that promote CENP-A^Cnp1^ incorporation. However, in FACT defective *spt16-18* cells the central domain loses its ability to preferentially attract CENP-A^Cnp1^ so that it is drawn away and accumulates on *pot1* ^+^ and *ura4* ^+^ *-int-cc2* at similar levels (Figure S6D). This implies that when FACT function is compromised some genes present the same features as those presented by the central domain regions so that they become equivalently competent in attracting excess CENP-A^Cnp1^. The central domain and subtelomeric regions must share specific features that attract CENP-A^Cnp1^ and both lose this exclusivity when FACT is defective.

**Figure 4:**
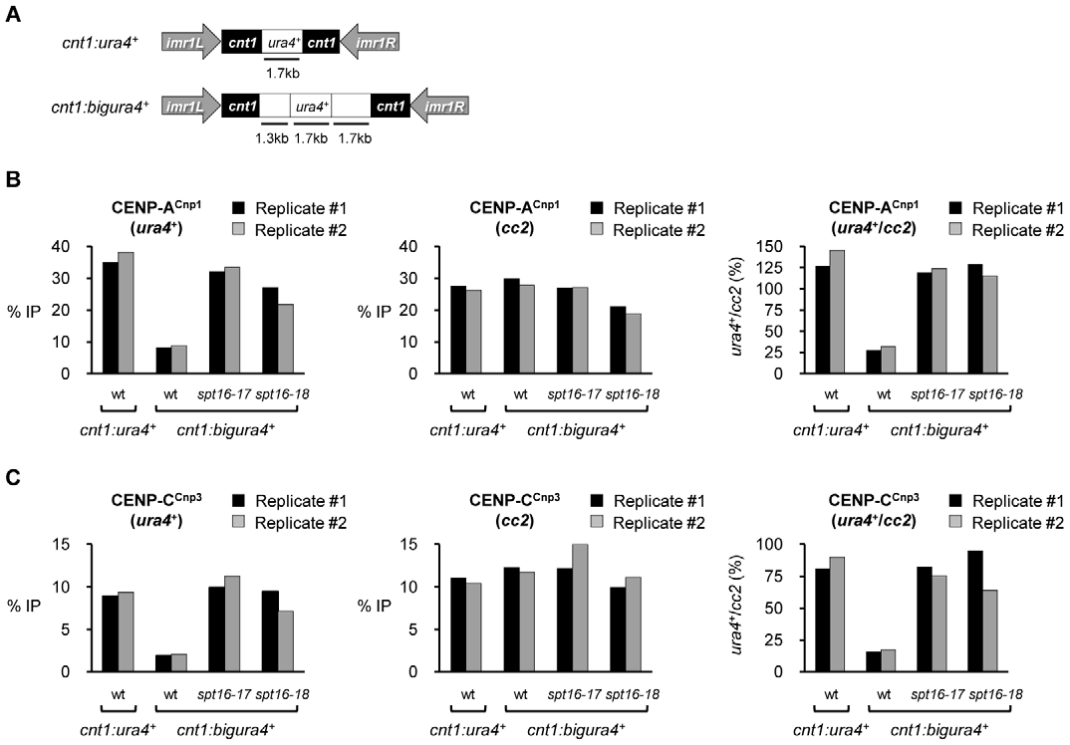
Spt16 prevents efficient assembly of CENP-A^Cnp1^ chromatin on large non-centromeric DNA inserted within the central domain. (A) Schematic of *cnt1:ura4* ^+^ and *cnt1:bigura4* ^+^. (B) ChIP analysis of CENP-A^Cnp1^ at *ura4* ^+^ and endogenous centromere (*cc2*) in the indicated strains. *ura4* ^+^/*cc2*: relative enrichment of CENP-A^Cnp1^ at *ura4* ^+^ compared to *cc2*. Cells were grown at 36°C for 1 h after shift from 25°C. (C) ChIP analysis of CENP-C^Cnp3^ in the same samples. Data for two biological replicates (#1 and #2) are presented. Enrichment is reported as % IP.

To further examine the role of FACT in restricting the sequences on which CENP-A^Cnp1^ is normally incorporated, we used strains with small (1.7 kb; *cnt1*:*ura4* ^+^) or large (4.7 kb; *cnt1*:*bigura4* ^+^) gene insertions of non-centromeric DNA in the central domain of *cen1* (Figure 4A) [38]. In wild-type cells expressing normal levels of CENP-A^Cnp1^, CENP-A^Cnp1^ was highly enriched on the small *ura4* ^+^ gene (*cnt1*:*ura4* ^+^; Figure 4B), whilst four-fold less CENP-A^Cnp1^ was detected over *ura4* ^+^ in *cnt1*:*bigura4* ^+^. Thus, the large non-centromeric DNA insertion is a relatively poor substrate for CENP-A^Cnp1^ deposition even though it is placed in an environment conducive for CENP-A^Cnp1^ assembly. However, in FACT defective cells increased CENP-A^Cnp1^ assembles on *cnt1*:*bigura4* ^+^; similar to the levels detected on the smaller *cnt1*:*ura4* ^+^ insertion or the endogenous *cen2* central domain (*cc2*). The relative enrichment of CENP-C^Cnp3^ on *ura4* ^+^ and *cc2* is essentially identical to that of CENP-A^Cnp1^ in all cases (Figure 4C). RT-PCR analysis shows that transcription of *ura4* ^+^ from *cnt1*:*ura4* ^+^ or *cnt1*:*bigura4* ^+^ is not significantly affected in *spt16-18* cells (Figure S7). This suggests that altered nucleosome dynamics rather than transcriptional activity causes increased CENP-A^Cnp1^ incorporation on *bigura4* ^+^ in FACT mutants. These analyses demonstrate that FACT usually acts to prevent the incorporation of CENP-A^Cnp1^ on non-centromeric DNA such as RNAPII genes and in its absence the sequence specificity for the assembly of CENP-A^Cnp1^ on central domain DNA and subtelomeric regions is abolished. The observation that FACT does not prevent CENP-A^Cnp1^ incorporation at endogenous *cen2* central domain suggests that FACT activity is normally inhibited or limited at native centromeres to favor CENP-A^Cnp1^ deposition within centromeric DNA. Similarly FACT activity must be excluded from, or counteracted in, subtelomeric regions to allow CENP-A^Cnp1^ incorporation.

### Stable maintenance of CENP-A^Cnp1^ chromatin at centromeres requires functional FACT

**Figure 5:**
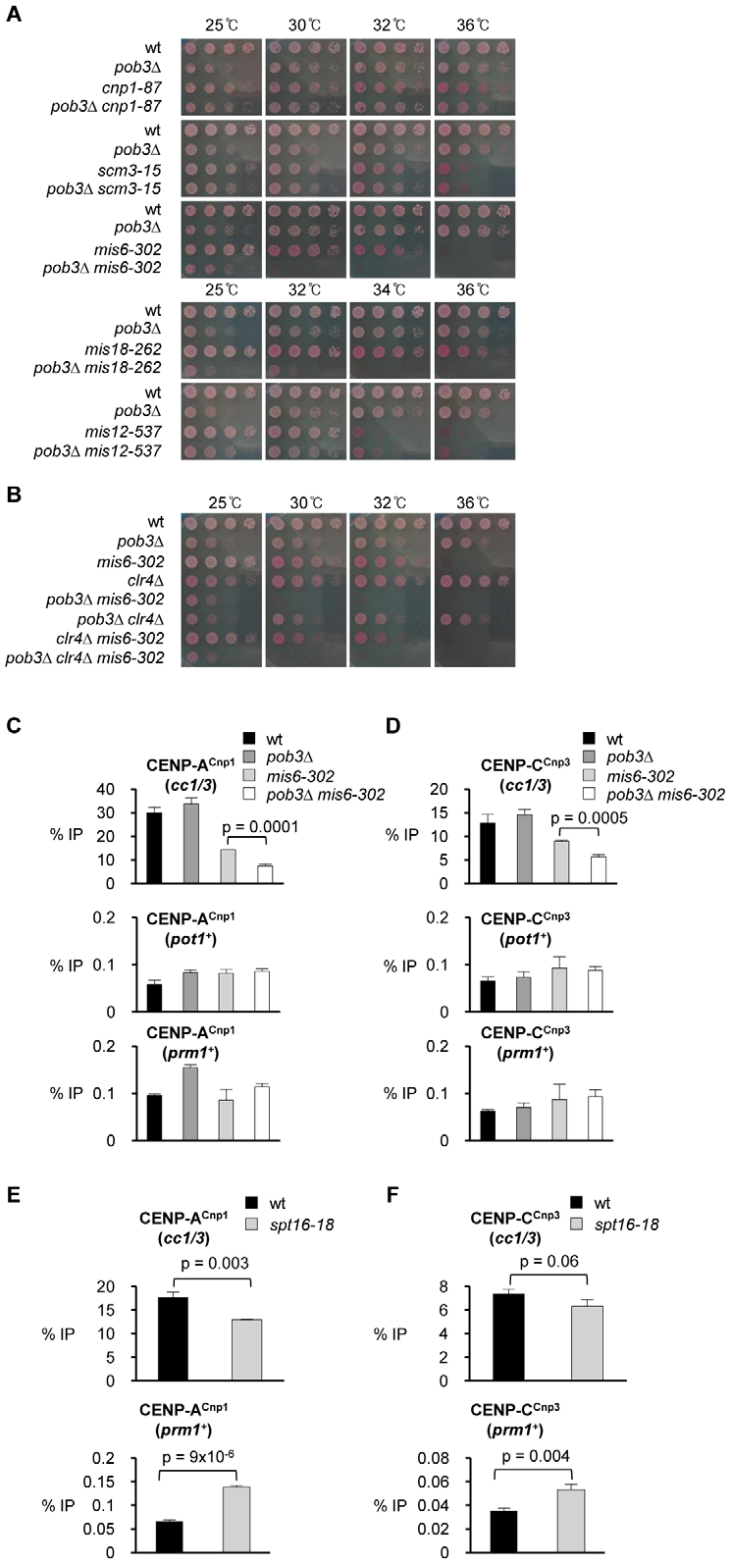
Pob3 genetically interacts with Mis6 and Mis18 and is required to maintain CENP-A^Cnp1^ at endogenous centromeres in *mis6-302* cells. (A) Viability of cells bearing *pob3*Δ combined with mutants affecting centromere function (*cnp1-87*, *scm3-15*, *mis6-302* and *mis12-537*) relative to wt cells and single mutants. Cells were spotted on plates containing Phloxine B at indicated temperatures. (B) Viability of wt and mutant cells bearing a combination of mutations in Pob3, Mis6 and Clr4 (*pob3*Δ, *mis6-302*, *clr4*Δ, *pob3*Δ *mis6-302*, *pob3*Δ *clr4*Δ, *clr4*Δ *mis6-302* and *pob3*Δ *clr4*Δ *mis6-302*). (C) ChIP analysis of CENP-A^Cnp1^ at endogenous centromeres (*cc1/3*), *pot1* ^+^ and *prm1* ^+^ in the indicated strains. Cells were grown at 25°C, shifted to 30°C for 17 h after shift from 25°C. (D) ChIP analysis of CENP-C^Cnp3^ in the same samples. (E) ChIP analysis of CENP-A^Cnp1^ levels at endogenous centromeres (*cc1/3*) and *prm1* ^+^ in wt and *spt16-18* cells grown at 27°C (semi-permissive for *spt16-18*) for 24 h. (F) ChIP analysis of CENP-C^Cnp3^ in the same samples. Enrichment is reported as % IP. Error bars indicate S.D. from 3 biological replicates.

Our above analyses indicate that FACT activity blocks the deposition of CENP-A^Cnp1^ at non-centromeric locations. However, CENP-A^Cnp1^ remains at centromeres in FACT defective cells and is not dispersed across the genome unless additional CENP-A^Cnp1^ is expressed (Figure 3A and 3D). Thus, a strong propagation mechanism must remain operational at centromeres to maintain CENP-A^Cnp1^ and prevent the redistribution of this limited CENP-A^Cnp1^ pool when FACT function is compromised. The Mis6 and Mis18 kinetochore proteins have been shown to be required to replenish CENP-A^Cnp1^ at centromeres [44], [45]. Mutations in Mis6 (*mis6-302*) or Mis18 (*mis18-262*) exhibited synthetic lethality when combined with a deletion of the gene encoding the small subunit FACT (*pob3*Δ) at semi-permissive temperatures (Figure 5A). In contrast, defective Mis12 (*mis12-537*), an essential kinetochore protein not involved in CENP-A^Cnp1^ maintenance [45], does not genetically interact with *pob3*Δ. In addition, mutations in CENP-A^Cnp1^ (*cnp1-87*) or its chaperone Scm3 (*scm3-15*) do not exhibit synergistic phenotypes in combination with *pob3*Δ since they must impair the deposition of CENP-A^Cnp1^ regardless of the genomic location [38], [46], [47]. We also found that deletion of the gene encoding Sim3 (*sim3*Δ), the NASP (N1/N2)-related CENP-A^Cnp1^ chaperone [48], relieves the lethal effect of CENP-A^Cnp1^ overexpression in FACT defective *pob3*Δ cells (Figure S8). This implies that Sim3 participates in the promiscuous deposition of CENP-A^Cnp1^ in FACT defective cells. Since Pob3 is also involved in heterochromatin integrity, the synergistic phenotype of *pob3*Δ *mis6-302* and *pob3*Δ *mis18-262* double mutants could be due to defective heterochromatin rather than altered CENP-A^Cnp1^ deposition [36]. However, this possibility is excluded because *mis6-302* does not display synergistic phenotypes when combined with *clr4*Δ which abolishes heterochromatin (Figure 5B).

Consistent with the observed genetic interaction between Mis6 and Pob3 mutants, the levels of CENP-A^Cnp1^ and CENP-C^Cnp3^ at centromeres were found to be significantly reduced in *pob3*Δ *mis6-302* cells compared to the *pob3*Δ or *mis6-302* single mutants (Figure 5C and 5D). Thus, when the mechanism for CENP-A^Cnp1^ maintenance at centromeres is compromised, CENP-A^Cnp1^ is released and the lack of FACT function allows its redistribution to non-centromeric sites. However, in *pob3*Δ *mis6-302* cells, the association of CENP-A^Cnp1^ with non-centromeric genes such as *pot1^+^* and *prm1* ^+^ is not elevated, probably because the redistribution and dilution of the limited endogenous pool of CENP-A^Cnp1^ over many non-centromeric sites is below the level of detectability. A low level of additional CENP-A^Cnp1^ (nmt81-CENP-A^Cnp1^) partially rescues the lethality of *pob3*Δ *mis6-302* cell at 32°C (Figure S9; Note: additional CENP-A^Cnp1^ reduces *pob3*Δ viability). This reinforces the conclusion that the observed synthetic lethality of the *pob3*Δ *mis6-302* double mutant cells results from reduced levels of CENP-A^Cnp1^ at centromeres.

Accumulation of CENP-A^Cnp1^ at non-centromeric regions was not detectable in FACT defective *spt16-18* cells expressing normal CENP-A^Cnp1^ levels after short-term inactivation (1 h at 37°C; Figure 3D). The redistribution of CENP-A^Cnp1^ in *spt16-18* cells may require a longer time period or cell cycle progression. To test this possibility, we examined the CENP-A^Cnp1^ redistribution in *spt16-18* cells expressing normal CENP-A^Cnp1^ levels after prolonged incubation at a semi-permissive temperature (24 h at 27°C). Under these conditions we detect reduced levels of CENP-A^Cnp1^ and CENP-C^Cnp3^ at centromeres and an increased association of both proteins with the *cen1* proximal gene *prm1* ^+^ (Figure 5E and 5F). Thus endogenous CENP-A^Cnp1^ can be redistributed from centromeres in cells not overexpressing CENP-A^Cnp1^ when FACT function is impaired for a sustained period. We conclude that FACT contributes to the stable maintenance of CENP-A^Cnp1^ chromatin at centromeres by preventing the aberrant deposition of CENP-A^Cnp1^ at non-centromeric locations.

### Loss of Clr6 complex II (Rpd3S) allows limited redistribution of CENP-A^Cnp1^

**Figure 6:**
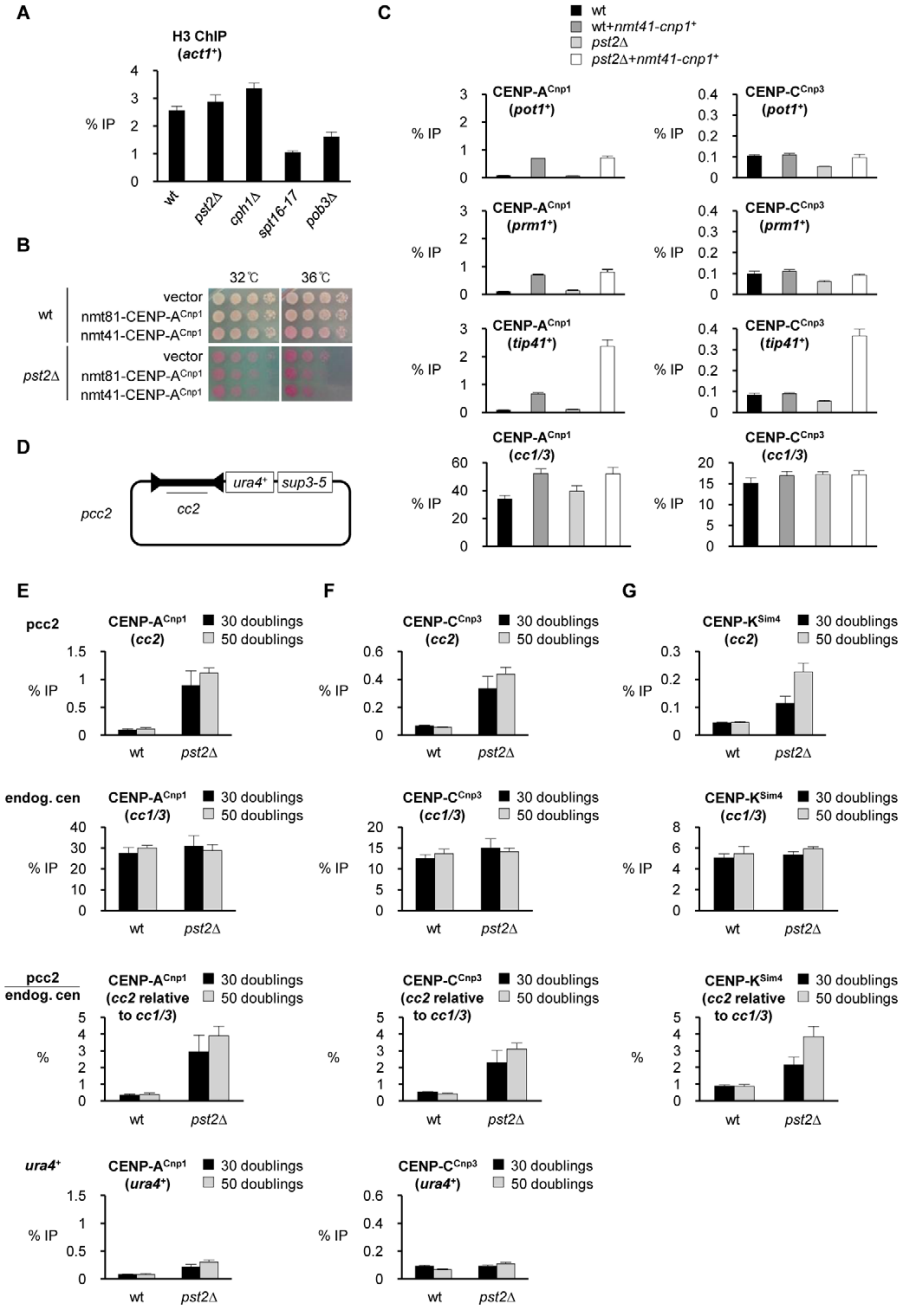
Loss of Clr6-CII function promotes assembly of CENP-A^Cnp1^ chromatin at specific loci. (A) ChIP analysis of H3 levels at *act1* ^+^ in the indicated strains grown at 32°C. Note: *spt16-17* (but not *spt16-18*) cells are viable at 32°C and thus are grown in parallel with other mutants as a positive control in this experiment. (B) Viability of wt and *pst2*Δ cells expressing additional CENP-A^Cnp1^ at low (nmt81-CENP-A^Cnp1^) or medium (nmt41-CENP-A^Cnp1^) levels compared to empty vector at 32°C and 36°C. (C) ChIP analyses of CENP-A^Cnp1^ and CENP-C^Cnp3^ levels in wt and *pst2*Δ cells at *pot1* ^+^, *prm1* ^+^, *tip41* ^+^ and endogenous centromeres (*cc1/3*). Cells were grown at 32°C. (D) Schematic of pcc2 plasmid. pcc2 plasmid contains 8.6 kb *cen2* central domain (*cc2*), *ura4* ^+^ and *sup3-5*. (E) ChIP analysis of CENP-A^Cnp1^ levels at *cc2* in pcc2 plasmid and at endogenous centromere (*cc1/3*) in wt and *pst2*Δ cells carrying pcc2. The relative enrichment of CENP-A^Cnp1^ at *cc2* compared to endogenous centromere (*cc1/3*) is presented (*cc2* relative to *cc1/3*). Enrichment of CENP-A^Cnp1^ at *ura4* ^+^ in pcc2 is also measured. (F) ChIP analysis of CENP-C^Cnp3^ levels in the same samples. (G) ChIP analysis of Sim4 levels in the same samples. ChIP was performed after 30 and 50 cell doublings at 32°C from the introduction of pcc2. Enrichment is reported as % IP. Error bars indicate S.D. from at least 3 biological replicates.

Our analyses indicate that both FACT and Clr6-CII are required to maintain chromatin integrity on transcribed genes, as indicated by the appearance of short aberrant intragenic transcripts, when their function is compromised (Figure 2B and 2C). To determine if Clr6-CII is also required to maintain H3 chromatin and prevent CENP-A^Cnp1^ mis-incorporation ChIP analysis was performed on cells lacking Clr6-CII subunits (*pst2*Δ and *cph1*Δ). Unlike FACT defective *spt16-18* cells, no reduction in H3 occupancy was observed on highly transcribed genes in *pst2*Δ cells compared to wild-type cells (Figure 6A and Figure S10A). Loss of Clr6-CII function may only affect H3 chromatin accessibility and not nucleosome occupancy. In *S. cerevisiae* lack of Rpd3S HDAC has also been found to affect chromatin integrity but not histone occupancy [31], [49]. Thus Clr6-CII and Rpd3S have a more subtle impact on the integrity of chromatin associated with RNAPII transcribed genes in both yeasts. Consistent with this, *S. pombe pst2*Δ cells are not hypersensitive to the expression of excess CENP-A^Cnp1^ (Figure 6B). Thus defective Clr6-CII may not induce the widespread mis-incorporation of CENP-A^Cnp1^ with associated loss of viability. Indeed in *pst2*Δ cells expressing additional CENP-A^Cnp1^, high levels of CENP-A^Cnp1^ and CENP-C^Cnp3^ were detected on *tip41* ^+^, but not on the *pot1* ^+^ or *prm1* ^+^ genes (Figure 6C). Furthermore, analysis of the genome-wide distribution of CENP-A^Cnp1^ in *pst2*Δ versus wild-type cells overexpressing CENP-A^Cnp1^ revealed that the mis-incorporation of CENP-A^Cnp1^ is far less prevalent in *pst2*Δ than *spt16-18* cells (Figure S10B). However, as with FACT defective *spt16-18* cells, the most notable accumulation of CENP-A^Cnp1^ in *pst2*Δ Clr6-CII deficient cells was observed over the euchromatic regions proximal to centromeres. Interestingly, a low but consistent increase in CENP-A^Cnp1^ levels also occurred over subtelomeric regions of chromosomes 1 and 2 where FACT was found not to affect CENP-A^Cnp1^ incorporation (Figure S10B and Figure 3C). It seems likely that Clr6-CII activity is generally not required to suppress replacement of H3 nucleosome with CENP-A^Cnp1^ nucleosome. However, in particular regions such as subtelomeric domains, where the activity of FACT in promoting H3 assembly appears limited, or in regions proximal to centromeres which are prone to CENP-A^Cnp1^ assembly; the activity of the Clr6-CII HDAC may normally be required to suppress histone exchange by transcription-coupled deacetylation of resident H3 nucleosomes.

### Clr6 Complex II (Rpd3S) prevents the assembly of CENP-A^Cnp1^ chromatin on centromeric central domain DNA

The endogenous levels of CENP-A^Cnp1^ in wild type cells are insufficient to permit its accumulation at non-centromeric regions. The expression of additional CENP-A^Cnp1^ allows its incorporation at more non-centromeric locations in cells with defective FACT or Clr6-CII. However, wild-type CENP-A^Cnp1^ levels are sufficient to allow CENP-A^Cnp1^ chromatin and functional kinetochores to be assembled *de novo* over extra centromeric central domain DNA introduced on plasmid-based minichromosomes. In these *de novo* establishment assays heterochromatin is normally required to promote the assembly of CENP-A^Cnp1^ chromatin on adjacent central domain DNA [10]. We tested if mutations that allow CENP-A^Cnp1^ incorporation at non-centromeric locations when CENP-A^Cnp1^ is overexpressed alter the requirements for *de novo* CENP-A^Cnp1^ assembly on centromeric DNA in the absence of CENP-A^Cnp1^ overexpression. Plasmids bearing only central domain DNA with no heterochromatin forming repeat sequences are usually unable to assemble CENP-A^Cnp1^ upon introduction into wild-type cells (pcc2; Figure 6D) [10]. Surprisingly, CENP-A^Cnp1^ and the kinetochore proteins CENP-C^Cnp3^ and CENP-K^Sim4^ were readily detected over the central domain of pcc2 following its transformation into *pst2*Δ cells (Figure 6E, 6F, 6G). This effect is specific for centromeric central domain DNA since CENP-A^Cnp1^ and CENP-C^Cnp3^ were not significantly enriched on the plasmid borne *ura4* ^+^ gene (Figure 6E and 6F). It is also specific to Clr6-CII mutants since CENP-A^Cnp1^ and CENP-C^Cnp3^ assemble on the central domain of pcc2 in cells lacking subunits of Clr6-CII/Rpd3S (*pst2*Δ and *cph1*Δ), but not Clr6-CI (*pst1-1*; Figure S10C). Defects in FACT/Spt16 or Spt6 function did not permit the assembly of CENP-A^Cnp1^ on pcc2 (Figure S10D and S10E). Importantly, ChIP analysis shows that *pst2*Δ does not induce H3K9 methylation on the pcc2 plasmid, indicating that the *de novo* CENP-A^Cnp1^ chromatin assembly pcc2 in *pst2*Δ cells is not induced by aberrant heterochromatin formation (Figure S11).

**Figure 7:**
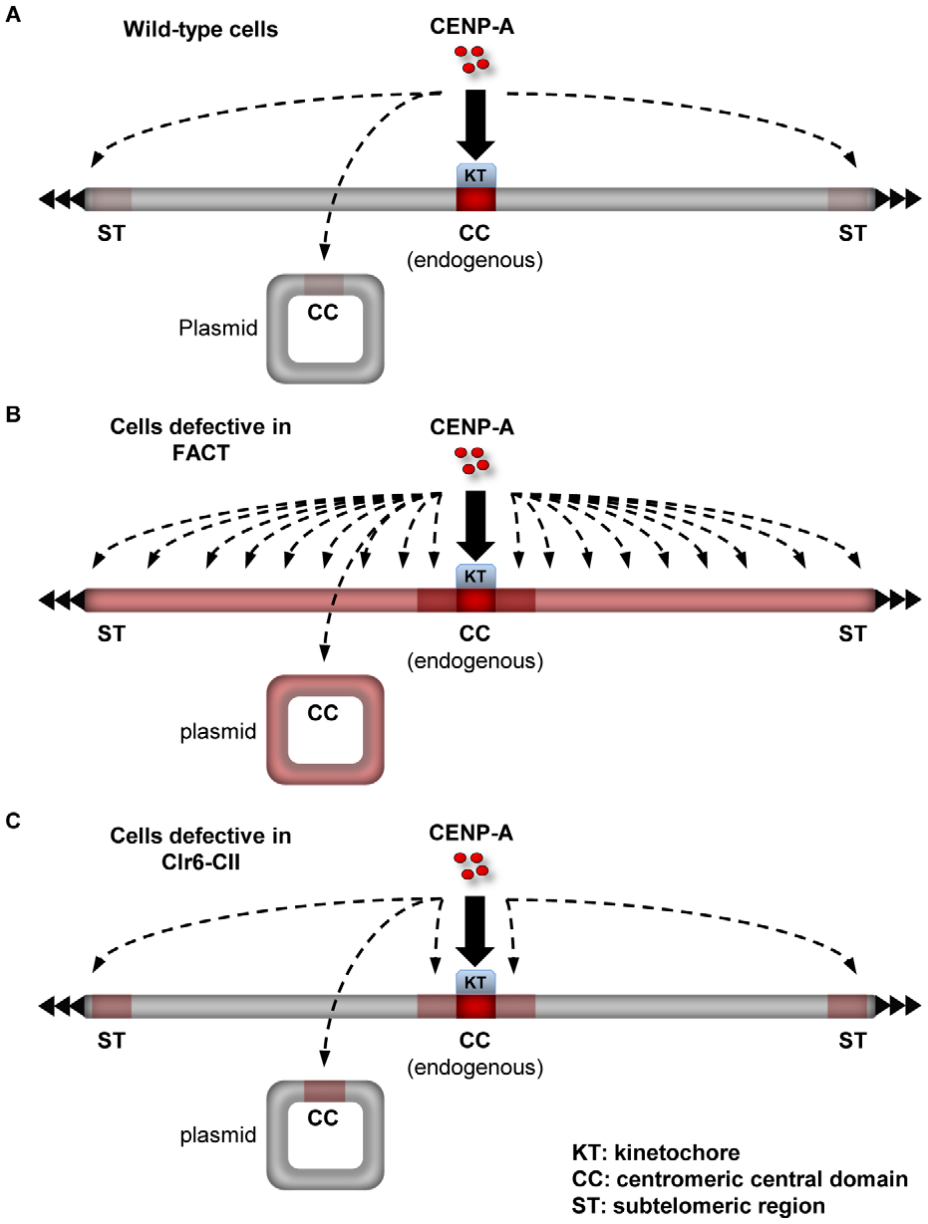
Summary on the role of factors that promote the integrity of H3 chromatin during transcription in preventing promiscuous CENP-A^Cnp1^ deposition. (A) In wild-type cells, a limited amount of free CENP-A^Cnp1^ is available to accumulate outside endogenous centromeres at which kinetochore proteins act to attract CENP-A^Cnp1^. This limited pool of free CENP-A^Cnp1^ can be preferentially deposited to specific sites such as centromeric central domain (CC) and subtelomeric regions (ST). Intensity of red color on the chromosome and the plasmid represents relative “receptiveness” of the locus for CENP-A^Cnp1^ incorporation. Bold arrow indicates regions where CENP-A^Cnp1^ incorporation normally occurs without overexpression (i.e. centromeres). Dashed arrows indicate regions where *de novo* assembly of CENP-A^Cnp1^ is expected under conditions where CENP-A^Cnp1^ deposition is stimulated (e.g. when CENP-A^Cnp1^ is overexpressed or flanking heterochromatin is provided). (B) In cells with defective FACT, non-centromeric regions become permissive to CENP-A^Cnp1^ (indicated by red color all over the chromosome and the plasmid). Endogenous centromeres drive CENP-A^Cnp1^ assembly at proximal euchromatic regions when they become permissive to CENP-A^Cnp1^ assembly (indicated by dark red color at centromere-proximal regions). CENP-A^Cnp1^ incorporation when overexpressed is not significantly elevated at CC and ST in FACT mutants compared to wild-type cells, suggesting that FACT action may be already limited at these sites. Without CENP-A^Cnp1^ overexpression, the limited pool of free CENP-A^Cnp1^ is distributed to non-centromeric regions and cannot accumulate at normally preferred sites such as CC and ST. (C) In cells with defective Clr6-CII, only specific regions such as centromere-proximal euchromatic regions, CC and ST become permissive to CENP-A^Cnp1^. Clr6-CII has a weaker impact on H3 chromatin compared to FACT and thus loss of Clr6-CII function promotes CENP-A^Cnp1^ incorporation only at regions where CENP-A^Cnp1^ assembly is predisposed (centromere-proximal regions) or FACT action is limited (CC and ST). In Clr6-CII mutants, limited pool of free CENP-A^Cnp1^ is not distributed and can accumulate at preferred sites, allowing *de novo* assembly of CENP-A^Cnp1^ chromatin on a plasmid bearing CC in the absence of flanking heterochromatin.

The distinct impact of mutations in Clr6-CII compared to FACT or Spt6 in the pcc2 *de novo* assembly assay is indicative of competition between the distinct genomic loci affected by these mutations for the limited pool of endogenous CENP-A^Cnp1^. Defective FACT function results in widespread loss of H3 chromatin and this renders a large fraction of the genome receptive for CENP-A^Cnp1^ incorporation. Under these circumstances the normal limited pool of free CENP-A^Cnp1^ is broadly distributed and CENP-A^Cnp1^ cannot be specifically recruited and assembled *de novo* on the newly introduced pcc2 (Figure 7A and 7B). In contrast, cells with defective Clr6-CII function have a more subtle alteration in H3 chromatin integrity and this allows CENP-A^Cnp1^ to be incorporated into particular regions that possess intrinsic properties which favor CENP-A^Cnp1^ deposition, such as pcc2 and subtelomeric regions (Figure 7C). We conclude that the defect in restoring H3 chromatin integrity after RNAPII transcription that results from loss of Clr6-CII function removes an impediment to the efficient CENP-A^Cnp1^ assembly onto its preferred substrate, central domain DNA, and this bypasses the need for flanking heterochromatin. It follows that flanking centromeric heterochromatin may impose particular constraints on chromatin disassembly/reassembly events associated with central domain transcription in order to reduce H3 nucleosome stability and promote CENP-A^Cnp1^ assembly in its place.

## Discussion

In most eukaryotic organisms, centromere identity is determined epigenetically. However, certain DNA elements such as human α-satellite repeats are the sites where endogenous centromeres are normally located [1], [5]. Moreover, the introduction of α-satellite repeat DNA allows the *de novo* assembly of functional centromeres [50]. Thus these repeat elements represent preferred substrates, suggesting that their underlying DNA sequence plays some role in specifying the location of centromeres [6]. Previously, we demonstrated that central domain DNA at normal centromeres in fission yeast is transcribed by RNAPII [24]. Here, we show that factors such as FACT and Clr6-CII, which actively promote the integrity of H3 chromatin during RNAPII transcription, suppress the incorporation of excess CENP-A^Cnp1^. Importantly, we find that FACT blocks the incorporation of CENP-A^Cnp1^ over large portions of the genome. However this restraint does not operate in genomic regions where CENP-A^Cnp1^ assembly is normally favored i.e centromeres and subtelomeric regions. These regions accumulate higher levels of CENP-A^Cnp1^ in Clr6-CII mutants, suggesting a role for transcription-coupled processes in CENP-A^Cnp1^ assembly at these sites. We propose a model in which certain DNA sequences and chromatin contexts have the ability to restrict FACT activity during transcription and consequently represent hotspots for CENP-A^Cnp1^ assembly (summarized in Figure 7).

### Competition between CENP-A^Cnp1^ and H3 for incorporation into chromatin

CENP-A^Cnp1^ competes with histone H3 for incorporation into centromeric chromatin [38]. At centromeres, mechanisms which ensure the maintenance of CENP-A^Cnp1^ act to prevent incorporation of H3. Similarly, we find that at non-centromeric regions, mechanisms that maintain H3 nucleosomes during the intrinsically disruptive process of transcription act to prevent incorporation of CENP-A^Cnp1^. FACT is involved in several chromatin-based processes in addition to transcription, and thus it is possible that promiscuous CENP-A^Cnp1^ incorporation in FACT mutants is caused by defects in other processes rather than transcription-coupled nucleosome reassembly [51]. Although this possibility cannot be ruled out completely, we have observed similar CENP-A^Cnp1^ phenotypes in cells with defective Spt6 (Figure S5A and S5B). This supports a direct connection between defective H3 chromatin assembly during RNAPII transcription and promiscuous CENP-A^Cnp1^ incorporation. We propose that CENP-A^Cnp1^ is opportunistic in nature and its assembly into chromatin is strongly affected by its availability relative to histone H3 and processes that promote transcription-coupled recycling of H3 nucleosomes. When excess free CENP-A^Cnp1^ is available and transcription-coupled H3 nucleosome assembly is defective, CENP-A^Cnp1^ gains access to regions from which it is normally excluded. In this context it should be noted that the N-terminal tail of CENP-A^Cnp1^ is distinct from that of H3; it lacks the key lysine residues, K4, K9, K14, K18, K23, K27, and K36. Thus, CENP-A^Cnp1^ is undoubtedly managed differently than H3 with respect to transcription.

What gives CENP-A^Cnp1^ a selective advantage for incorporation into non-centromeric regions in FACT defective (*spt16-18*) cells? Our observation that H3 occupancy decreases in *spt16-18* cells in proportion to transcription rates suggests that H3 incorporation from the free histone pool is not sufficiently effective to maintain a steady-state level of H3 on transcribed chromatin templates in *spt16-18* cells (Figure 2F). Thus, the maintenance of H3 chromatin within transcribed regions must be largely dependent on replication-coupled assembly in S phase and histone chaperone-mediated reassembly during transcription. It is known that CENP-A incorporation at centromeres is uncoupled from replication in human cells and in fission yeast CENP-A incorporation can occur in S phase or in G2 phase independently of replication [48], [52]. It therefore seems likely that transcription-coupled loss of H3 nucleosomes in *spt16-18* cells may favor the incorporation of CENP-A. Given the differences between CENP-A and H3, CENP-A nucleosomes are likely to react differently to transcription and persist in situations that cause H3 nucleosomes to disassemble during transcription. The HDAC Clr6-CII preferentially targets RNAPII-transcribed regions [33]. In cells that are defective in Clr6-CII function (*pst2*Δ, *cph2*Δ), the persistence of transcription-associated histone acetylation destabilizes H3 nucleosomes (as indicated by the exposure of cryptic transcription initiation sites) and thereby enhances their replacement with CENP-A^Cnp1^ nucleosomes. The distinct N-terminal tail of CENP-A^Cnp1^ presumably provides CENP-A^Cnp1^ nucleosomes with greater stability than H3 nucleosomes during transcription in Clr6-CII mutant cells. It is also possible that once assembled, CENP-A^Cnp1^ nucleosomes repress transcription and that this reduces nucleosome turnover and consequently stabilizes CENP-A^Cnp1^ chromatin. Further analyses are required to reveal exactly how CENP-A replaces H3 and is stabilized on transcription units.

### Genetic and epigenetic processes influencing CENP-A^Cnp1^ chromatin assembly

The data that we present suggest that defective FACT function diminishes the distinction between centromeric and non-centromeric regions, allowing widespread incorporation of CENP-A^Cnp1^ into transcribed DNA when CENP-A^Cnp1^ is overexpressed. The central domains from centromeres (ectopically placed *cc2* DNA; Figure S6C) and potential sites of neocentromeres in subtelomeric regions (Figure S6A) clearly have an innate ability to incorporate CENP-A^Cnp1^ in wild-type cells that express additional CENP-A^Cnp1^. However, in FACT mutants, many other genomic locations become permissive for CENP-A^Cnp1^ incorporation, therefore the pool of additional free CENP-A^Cnp1^ is distributed over many chromosomal regions so that preferential incorporation at normal secondary sites such as ectopic *cc2* or subtelomeric regions is reduced (Figure 3C and Figure S6D). We conclude that central domain and subtelomeric regions, which naturally favor CENP-A^Cnp1^ deposition, possess features that reduce or evade FACT action so that H3 is more readily replaced by CENP-A^Cnp1^. Consistent with a role for cis-acting elements, FACT does not suppress CENP-A^Cnp1^ assembly on the central domain DNA at endogenous centromeres while it does prevent efficient CENP-A^Cnp1^ assembly on a large non-centromeric reporter gene insertion at the endogenous centromere (Figure 4A, 4B, 4C).

Genetic interactions exhibited between Pob3 and Mis6 or Mis18, along with ChIP analyses, demonstrate that FACT operates to retain CENP-A^Cnp1^ at centromeres when the CENP-A^Cnp1^ maintenance mechanism is weakened because it prevents CENP-A^Cnp1^ incorporation elsewhere (Figure 5A, 5B, 5C, 5D). However, even in the presence of an intact CENP-A^Cnp1^ maintenance mechanism, prolonged attenuation of FACT/Spt16 function causes redistribution of CENP-A^Cnp1^ and CENP-C^Cnp3^ (Figure 5E and 5F). Thus, the CENP-A^Cnp1^ maintenance mechanism operated by the kinetochore is not sufficient to allow CENP-A^Cnp1^ maintenance at centromeres when FACT function is impaired for long periods; under these conditions, the sequence-driven preference for CENP-A^Cnp1^ assembly at centromeres is compromised.

### Perspective

Human FACT has been shown to interact with CENP-A nucleosomes [42]. In chicken DT40 cells FACT has been shown to be required for the deposition of newly synthesized CENP-A, but not for the maintenance of pre-existing CENP-A, at centromeres [53]. Although we cannot exclude a direct role for FACT in CENP-A chromatin assembly at centromeres, our analyses in *S. pombe* suggests that defective FACT function indirectly affects CENP-A^Cnp1^ deposition at centromeres by allowing CENP-A^Cnp1^ mis-incorporation at non-centromeric locations. This raises the possibility that the depletion of FACT in vertebrate cells may result in newly synthesized CENP-A being dispersed throughout the genome so that its incorporation at centromeres is reduced. In this regard, we predict that factors such as FACT, which are involved in transcription-coupled chromatin reassembly, will have a conserved role in preventing the mis-incorporation of CENP-A at non-centromeric locations in higher eukaryotes. It is possible that at centromeres CENP-A sequesters/inhibits FACT to reduce its activity in recycling H3 nucleosomes during RNAPII transcription through centromeric chromatin. Spt16 is known to directly bind the N-terminal tails and globular core domain of H3 [54]; it will be interesting to determine if CENP-A^Cnp1^ competes with H3 for binding to Spt16.

In *C. elegans*, CENP-A and centromere activity is distributed along chromosomes. Recent analyses show that transcription in the germline acts to exclude CENP-A incorporation in progeny [55]. In contrast, RNAPII/transcription has been found to be essential for the efficient binding of CENP-C and normal mitotic kinetochore function in human cells [56]. Our analyses in *S. pombe* suggest a model that reconciles these apparently disparate findings; RNAPII transcription may normally prevent CENP-A deposition at genes through the action of H3 nucleosome reassembly machineries such as FACT, however, when FACT function is defective, RNAPII transcription may promote CENP-A deposition. Thus, RNAPII transcription may act either positively or negatively on CENP-A deposition depending on the functionality of FACT. In monocentric organisms, it is possible that the function of FACT or other H3 nucleosome reassembly pathways is limited at centromeres so that RNAPII transcription at centromeres promotes CENP-A deposition.

Neocentromeres are rare in most systems, but they can form at novel locations in both natural and experimental situations [4], [12], [57] Given the link between transcription-coupled nucleosome dynamics and CENP-A^Cnp1^ assembly highlighted here, it is possible that neocentromeres tend to arise at locations where H3 nucleosomes are less robustly maintained, so that CENP-A and other histones are more frequently incorporated from the free pool, rather than being recycled. Telomeric chromatin may affect the dynamics of H3 nucleosomes on sub-telomeric transcription units so that they are particularly prone to replacement with CENP-A. Likewise, at centromeres heterochromatin may impose constraints on H3 nucleosomes stability during transcription to promote its replacement with CENP-A. The elevated levels of CENP-A, caused by loss of regulation in cancer cells, may increase the frequency at which CENP-A chromatin is established, inducing additional neocentromere formation with resulting in genome instability [14], [17].

## Materials and Methods

### Cell growth and manipulation

Standard genetic and molecular techniques were followed. Fission yeast methods were as described [58]. For the strains used in the experiments, see Table S2. The *cnt1*:*bigura4* ^+^ strain contains the *ura4* ^+^ embedded within additional DNA consisting of *ade6* ^+^ sequences inserted within the central domain of *cen1* [38]. Note: The sequence of the *ade6* ^+^ and *ura4* ^+^ genes is 61% A/T which is close to the average A/T content of 64% for the *S. pombe* genome.

### ChIP

ChIP was performed as described using anti-H3 antibody (ab1791, Abcam), anti-FLAG M2 affinity gel (F2426, Sigma), anti-CENP-A^Cnp1^ antibody, anti-CENP-C^Cnp3^ antibody and anti-H3K9me2 antibody and subsequently analyzed by quantitative PCR (qPCR) [59]. For primers used in qPCR, see Table S3.

### Growth of cells overexpressing CENP-A^Cnp1^ for ChIP analyses

Cells expressing additional CENP-A^Cnp1^ from integrated pREP41-*cnp1^+^* (*nmt41-CENP-A^Cnp1^*) or cells with integrated empty vector were initially grown in rich medium which contains thiamine to repress the expression of additional CENP-A^Cnp1^. The cells were then streaked on minimal (PMG) plates which lack thiamine to allow expression of *nmt41-CENP-A^Cnp1^*. Subsequently, cells were grown in PMG liquid medium (without thiamine) at 25°C and shifted to 36°C for 1 h to inactivate Spt16 function before ChIP analyses.

### Plasmid-based assay for assembly of CENP-A^Cnp1^ chromatin

A plasmid (pcc2) carrying central domain sequence (*cc2*) but not outer repeat sequence (*otr*) is introduced into wild-type, *pst2*Δ, *cph1*Δ, *pst1-1*, *spt16-6* or *spt6-1* cells by electroporation. Transformants were selected on PMG-ura plates supplemented with low adenine (1/50^th^) at 32°C which allow to distinguish cells with episomal plasmids from those containing integrated plasmids by the colony color (cells with integrated plasmids form white colonies whereas those with episomal plasmids form light pink colonies). The resulting transformants were grown in PMG-ura liquid medium and analyzed by ChIP-qPCR. To confirm that cells maintain episomal plasmids and do not accumulate integrated plasmids, a plasmid stability test was performed at the time of fixation. Cells (200∼2000) were plated onto PMG-ura supplemented with low adenine (1/50^th^) and allowed to form colonies. Samples exhibiting less than 2% of integrations (i.e. white colonies) were used for ChIP. To extend the number of cell doublings (to 50 doublings) in wild-type or *pst2*Δ cells carrying pcc2 without increasing the proportion of cells with integrated plasmid, the cells grown in PMG-ura liquid medium (30 doublings after transformation) were plated onto PMG-ura supplemented with low adenine (1/50^th^). Colonies with light pink color which maintain episomal plasmids without integration were selected and pooled together (∼200 colonies in total) in PMG-ura liquid medium. Cells were grown for additional 8 h (50 doublings after transformation) and subject to ChIP analyses. To confirm that cells maintain episomal plasmids and do not accumulate integrated plasmids, a plasmid stability test was performed at the time of fixation as described above.

### Generation of temperature sensitive alleles of *spt16* ^+^

To screen *spt16-ts* alleles, DNA fragment containing either 5′ or 3′ half of *spt16* ^+^ ORF was mutagenized in vitro using Gene Morph II random mutagenesis kit (Stratagene). Each end of the mutagenized fragments was fused with a kanMX6 marker gene or the upstream (for 5′ half; *spt16-1* to *spt16-12*)/downstream sequences (for 3′ half; *spt16-13* to *spt16-25*) of *spt16* ^+^ ORF by fusion PCR. The resulting fusion PCR products were further amplified using nested primers and introduced into wild-type cells by electroporation. Transformants were selected on plates containing G418 and temperature sensitive (*ts*) mutants were identified among G418-resistant colonies by lethality at 36°C after replica-plating to plates containing Phloxine B. To confirm that the temperature sensitivity is caused by mutations in *spt16* ^+^, a plasmid rescue experiment was performed. Mutants whose *ts* phenotypes are rescued by plasmid expressing wild-type *spt16* ^+^ were selected for further analyses and the causative mutations were identified by sequencing. For detailed information on the *spt16-ts* alleles, see Table S4.

### Northern analysis

Northern analysis was performed as described previously using in vitro transcribed RNA probes [24]. For details on the primers used to create the probes, see Table S3.

### Western analysis

Western analysis was performed as described previously using anti-GFP antibody (gift from Kevin Hardwick) and anti-TAT-1 antibody (alpha-tubulin - gift from Keith Gull) [47]. The intensities of GFP and TAT-1 signals were quantified using LICOR Odyssey Infrared Imaging System software (Li-COR Bioscience).

### ChIP–chip

DNA was immunoprecipitated as described earlier using 10 µl of anti-CENP-A^Cnp1^ and 1.5 µg of anti-H3 (ab1791, abcam) antibody per 100 µl chromatin extract [60]. For microarrays with spiked-in controls Affymetrix GeneChip Eukaryotic Poly-A RNA Control Kit was used. RNA from the kit was transcribed into cDNA using SuperScript II Reverse Transcriptase (invitrogen) and oligo(dT) primers (invitrogen). The cDNA was diluted 10,000 times and added to the immunoprecipitated samples before round A and B amplification. Fragmentation, labeling and hybridization to the Affymetrix GeneChip *S. pombe* Tiling 1.0FR was performed by Affymetrix core facility at Novum (BEA) according to Affymetrix standard protocols. Raw data from Affymetrix (.CEL format) were normalized with Affymetrix Tiling Analysis Software (TAS) v1.1 and analyzed and visualized using Podbat [61].

### RT–PCR analysis

RT–PCR using total RNAs prepared with RNeasy mini kit (Qiagen) was performed as described [24].

## Supporting Information

Figure S1 FACT is not required for central core silencing and does not show genetic interaction with CENP-A^Cnp1^. (A) Schematic of targeted random mutagenesis in 5′ or 3′ regions within *spt16* ^+^. See additional details in Materials and Methods. (B) Viability of wt, *cnp1-87* and *spt16-ts* cells with *cnt1*:*ura4* ^+^ on N/S (non-selective), -Ura (uracil lacking) and FOA (counterselective drug for *ura4* ^+^ expression) plates at 25°C. (C) Viability of wt, *pob3*Δ and *cnp1-87* cells with *cnt1*:*ura4* ^+^ on N/S (non-selective), -Ura and FOA plates at 32°C. (D) Viability of wt, *pob3*Δ, *cnp1-87* and *pob3*Δ *cnp1-87* cells at indicated temperatures.
(TIF)

Figure S2 CENP-A^Cnp1^ accumulates preferentially at centromere proximal regions in *spt16-18* cells with overexpression of CENP-A^Cnp1^. ChIP-chip analyses of relative levels of CENP-A^Cnp1^ in *spt16-18* cells compared to wt in the presence of OE-CENP-A^Cnp1^ (*nmt41-cnp1* ^+^) at centromere proximal regions. ORFs are displayed as grey boxes. Regions of at least 1 kb in length and with >2-fold increase in CENP-A^Cnp1^ signal above genome-wide average are depicted with red boxes. Data on the Y-axis are presented in linear scale. Blue: running average signal/100 probes. Grey: signal for individual probes.
(TIF)

Figure S3 Detection of cryptic shorter transcripts from *prm1* ^+^, *tip41* ^+^ and *act1* ^+^. (A) Northern analyses of transcripts from *prm1* ^+^ and *tip41* ^+^ gene. (B) Northern analyses of transcripts from *act1* ^+^ gene. RNA was extracted from cells grown at 25°C (wt, *spt16-18*), 32°C for 6 h (wt, *pst2*Δ) or 36°C for 1 h (wt, *spt16-18*) after shift from 25°C. Arrow indicates full-length transcripts.
(TIF)

Figure S4 H3 and CENP-A^Cnp1^ are preferentially incorporated in genes expressed at low to intermediate levels in *spt16-18* cells. Moving average plots (window size = 100, step size = 1) of H3 (upper panel) and CENP-A^Cnp1^ (lower panel) plotted as a function of RNA expression in WT (arbitrary units a.u.). H3/CENP-A^Cnp1^ association in *spt16-18* cells (red) and WT (black) at 36°C.
(TIF)

Figure S5 Effects of CENP-A^Cnp1^ overexpression in cells with defective Spt6. (A) Viability of wt, *spt6-1* and *spt16-20* cells expressing additional CENP-A^Cnp1^ at low (nmt81-CENP-A^Cnp1^) and medium (nmt41-CENP-A^Cnp1^) levels compared to empty vector at 32°C. Note: *spt16-20* cells have a semi-permissive temperature similar to that of *spt6-1* and thus are used as a positive control in this experiment. (B) ChIP analysis of CENP-A^Cnp1^ levels at *prm1* ^+^, *tip41* ^+^ and endogenous centromeres (*cc1/3*) in wt and *spt6-1* cells in the absence or presence of OE-CENP-A^Cnp1^ (*nmt41-cnp1* ^+^). Cells were grown at 36°C for 1 h after shift from 25°C.
(TIF)

Figure S6 CENP-A^Cnp1^ preferentially accumulates at subtelomeric regions and ectopically placed central domain DNA when overexpressed. (A) ChIP-chip analyses of relative levels of CENP-A^Cnp1^ in wt cells with OE-CENP-A^Cnp1^ (*nmt41-cnp1* ^+^) compared to wt cells without OE-CENP-A^Cnp1^. Cells were grown at 36°C for 1 h after shift from 25°C. Data on the Y-axis are presented in log2 scale. (B) Schematic of ectopic *cc2* inserted at *ura4* ^+^ locus (*ura4* ^+^ *-int-cc2*). (C) ChIP analysis of CENP-A^Cnp1^ levels at *act1* ^+^, *pot1* ^+^ and *ura4* ^+^ *-int-cc2* in wt cells in the absence or presence of OE-CENP-A^Cnp1^ (*nmt41-cnp1* ^+^) grown at indicated temperatures. (D) ChIP analysis of CENP-A^Cnp1^ levels at *act1* ^+^, *pot1* ^+^ and *ura4* ^+^ *-int-cc2* in wt and *spt16-18* cells in the absence or presence of OE-CENP-A^Cnp1^ (*nmt41-cnp1* ^+^). Cells were grown at 36°C for 1 h after shift from 25°C.
(TIF)

Figure S7 Expression of *ura4* ^+^ from *cnt1*:*ura4* ^+^ or *cnt1*:*bigura4* ^+^ is not significantly affected in *spt16-18* cells. qRT-PCR analyses to measure the levels of *ura4* ^+^ transcripts from *cnt1*:*ura4* ^+^ or *cnt1*:*bigura4* ^+^ in wild-type and *spt16-18* cells. Cells were grown at 36°C for 1 h after shift from 25°C. The relative expression levels were calculated as the value of *ura4* ^+^ expression relative to *act1* ^+^. These values (*ura4* ^+^/*act1* ^+^) were further normalized to those of respective wild-type (relative to wt). Error bars indicate S.D. from 2 biological replicates.
(TIF)

Figure S8 Loss of Sim3 relieves the toxic effects of CENP-A^Cnp1^ overexpression in *pob3*Δ cells. Viability of wt, *pob3*Δ, *sim3*Δ and *pob3*Δ *sim3*Δ cells expressing additional CENP-A^Cnp1^ at low (nmt81-CENP-A^Cnp1^) or medium (nmt41-CENP-A^Cnp1^) levels compared to empty vector. Cells were grown at 25°C, 32°C or 36°C. Phloxine B plates stain dead cells red.
(TIF)

Figure S9 Low level overexpression of CENP-A^Cnp1^ partially rescues the lethality of *pob3*Δ *mis6-302* cells. Viability of wt, *pob3*Δ, *mis6-302*, *pob3*Δ *mis6-302* and *cnp1-1* strains expressing additional CENP-A^Cnp1^ at medium (nmt41-CENP-A^Cnp1^) or low (nmt81-CENP-A^Cnp1^) levels compared to empty vector at 25°C or 32°C.
(TIF)

Figure S10 Defective function of Clr6-CII allows assembly of CENP-A^Cnp1^ chromatin at specific locations. (A) Average gene analysis for the ratio of H3 occupancy in *pst2*Δ mutants versus wt. Genes are aligned at transcription start site and divided into four groups dependent of their transcription levels. n = number of genes in each group. Error bars represent 99% confidence intervals. (B) ChIP-chip analyses of relative CENP-A^Cnp1^ levels in *pst2*Δ cells compared to wt in the presence of OE-CENP-A^Cnp1^ (*nmt41-cnp1* ^+^). ORFs are displayed as grey boxes. Regions of at least 1 kb in length and with >2-fold increase in CENP-A^Cnp1^ signal above genome-wide average are colored red. Data on the Y-axis are presented in linear scale. Blue: running average signal/100 probes. Grey: signal for individual probes. (C) ChIP analyses of CENP-A^Cnp1^ and CENP-C^Cnp3^ levels at *pot1* ^+^ and *cc2* in pcc2 plasmid compared to endogenous centromere (*cc1/3*) in wt, *pst2*Δ, *cph1*Δ and *pst1-1* cells carrying pcc2. Cells were collected after 30 cell doublings at 32°C from the introduction of pcc2. Error bar indicates standard deviation from 3-4 independent biological experiments. (D) ChIP analyses of CENP-A^Cnp1^ and CENP-C^Cnp3^ levels at *pot1* ^+^ and *cc2* in pcc2 plasmid compared to endogenous centromere (*cc1/3*) in wt and *spt16-6* cells carrying pcc2. Error bar indicates standard deviation from 3 independent biological experiments. (Note: we find that most of *spt16-ts* alleles including *spt16-18* do not allow efficient propagation of pcc2 plasmid and thus a specific allele (*spt16-6*) which allows propagation of pcc2 is used in this particular assay.) (E) ChIP analyses of CENP-A^Cnp1^ and CENP-C^Cnp3^ levels at *pot1* ^+^ and *cc2* in pcc2 plasmid compared to endogenous centromere (*cc1/3*) in wt and *spt6-1* cells carrying pcc2. Error bar indicates standard deviation from 3 independent biological experiments.
(TIF)

Figure S11 Loss of Clr6-CII function does not induce H3K9 methylation on pcc2 plasmid. (A) Schematic of pcc2 plasmid. Regions amplified by primer pairs used in ChIP-qPCR (*cc2*, *ura4* ^+^ and vector - a region on the plasmid backbone) are indicated as short black bars. (B) ChIP analyses of H3K9 methylation (H3K9me2) levels at *cc2*, *ura4* ^+^ and vector in pcc2 in wt and *pst2*Δ cells carrying pcc2. (C) ChIP analyses of H3K9me2 levels at chromosomal *act1* ^+^ and *dg* in the same samples. *dg* represents a part of heterochromatic centromere outer repeats and thus serves as a positive control for H3K9me2 ChIP. ChIP was performed after 30 and 50 cell doublings at 32°C from the introduction of pcc2. Enrichment is reported as % IP. Error bars indicate S.D. from 4 biological replicates.
(TIF)

Table S1 Relative enrichment of CENP-A^Cnp1^ and H3 in *spt16-18* versus wild-type cells (at 36°C) at selected genes from ChIP-chip data and their relative RNA expression levels (at 30°C; transcription levels were categorized as in Figure 2F).
(DOC)

Table S2 List of strains.
(DOC)

Table S3 List of primers.
(DOC)

Table S4 List of *spt16-ts* alleles.
(DOC)
