# Supplementary figures and images for: Factors That Promote H3 Chromatin Integrity during Transcription Prevent Promiscuous Deposition of CENP-ACnp1 in Fission Yeast
Source: PLoS Genet. 2012 Sep 20;8(9):e1002985. doi: 10.1371/journal.pgen.1002985 (PMC3447972; doi:10.1371/journal.pgen.1002985)

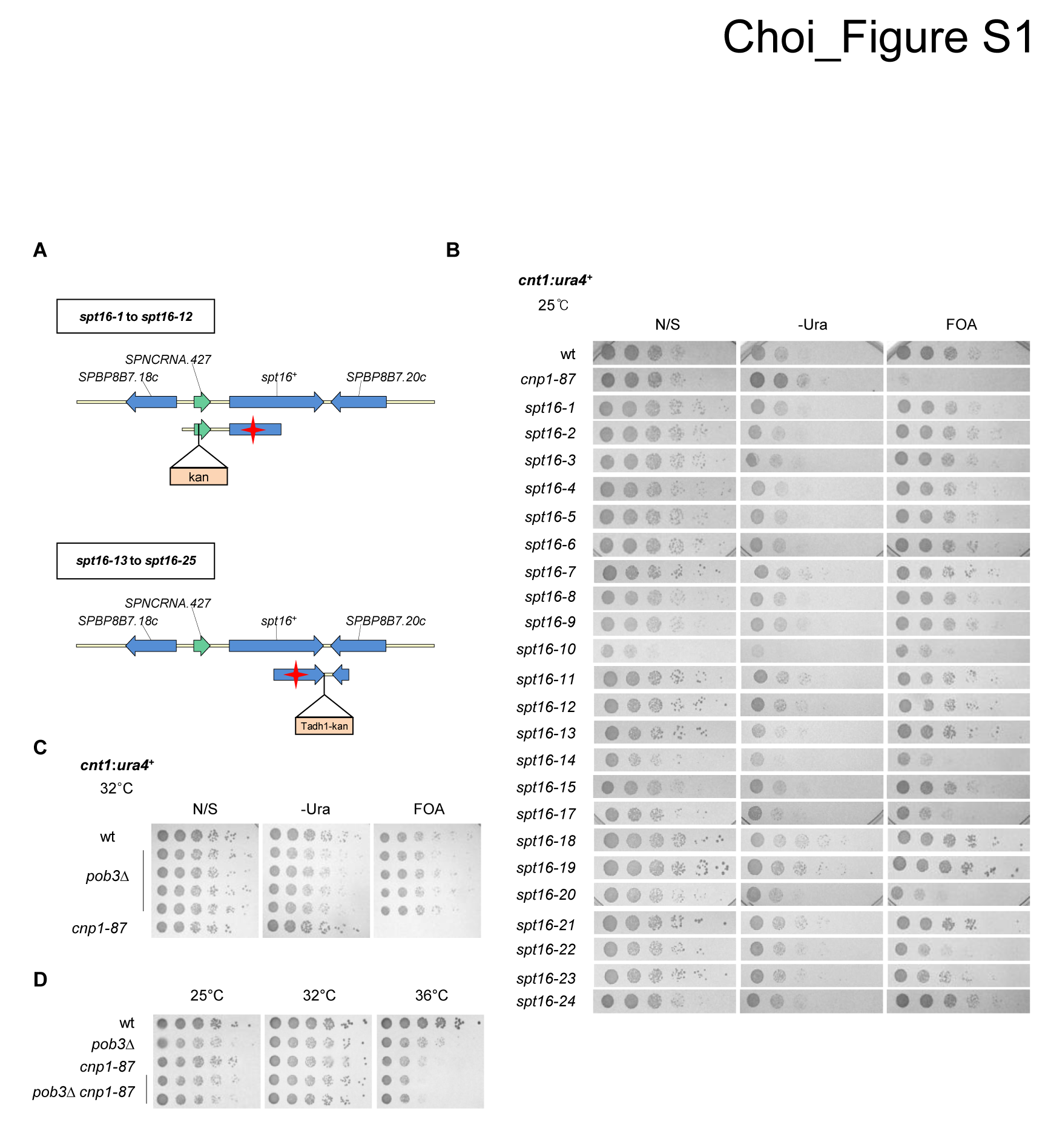

Supplement: Figure S1 — FACT is not required for central core silencing and does not show genetic interaction with CENP-ACnp1. (A) Schematic of targeted random mutagenesis in 5′ or 3′ regions within spt16 +. See additional details in Materials and Methods. (B) Viability of wt, cnp1-87 and spt16-ts cells with cnt1:ura4 + on N/S (non-selective), -Ura (uracil lacking) and FOA (counterselective drug for ura4 + expression) plates at 25°C. (C) Viability of wt, pob3Δ and cnp1-87 cells with cnt1:ura4 + on N/S (non-selective), -Ura and FOA plates at 32°C. (D) Viability of wt, pob3Δ, cnp1-87 and pob3Δ cnp1-87 cells at indicated temperatures. (TIF) [file pgen.1002985.s001.tif]

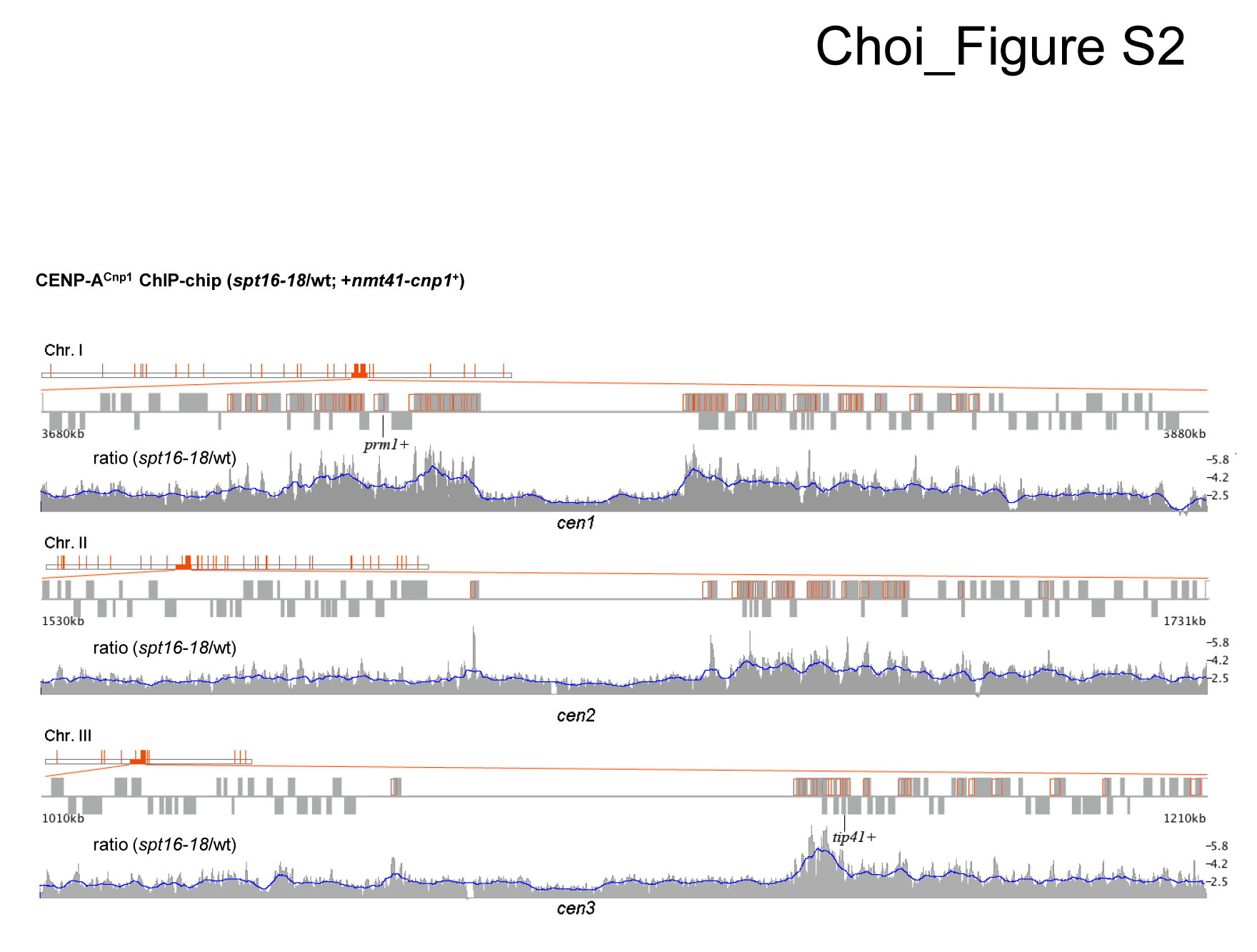

Supplement: Figure S2 — CENP-ACnp1 accumulates preferentially at centromere proximal regions in spt16-18 cells with overexpression of CENP-ACnp1. ChIP-chip analyses of relative levels of CENP-ACnp1 in spt16-18 cells compared to wt in the presence of OE-CENP-ACnp1 (nmt41-cnp1 +) at centromere proximal regions. ORFs are displayed as grey boxes. Regions of at least 1 kb in length and with >2-fold increase in CENP-ACnp1 signal above genome-wide average are depicted with red boxes. Data on the Y-axis are presented in linear scale. Blue: running average signal/100 probes. Grey: signal for individual probes. (TIF) [file pgen.1002985.s002.tif]

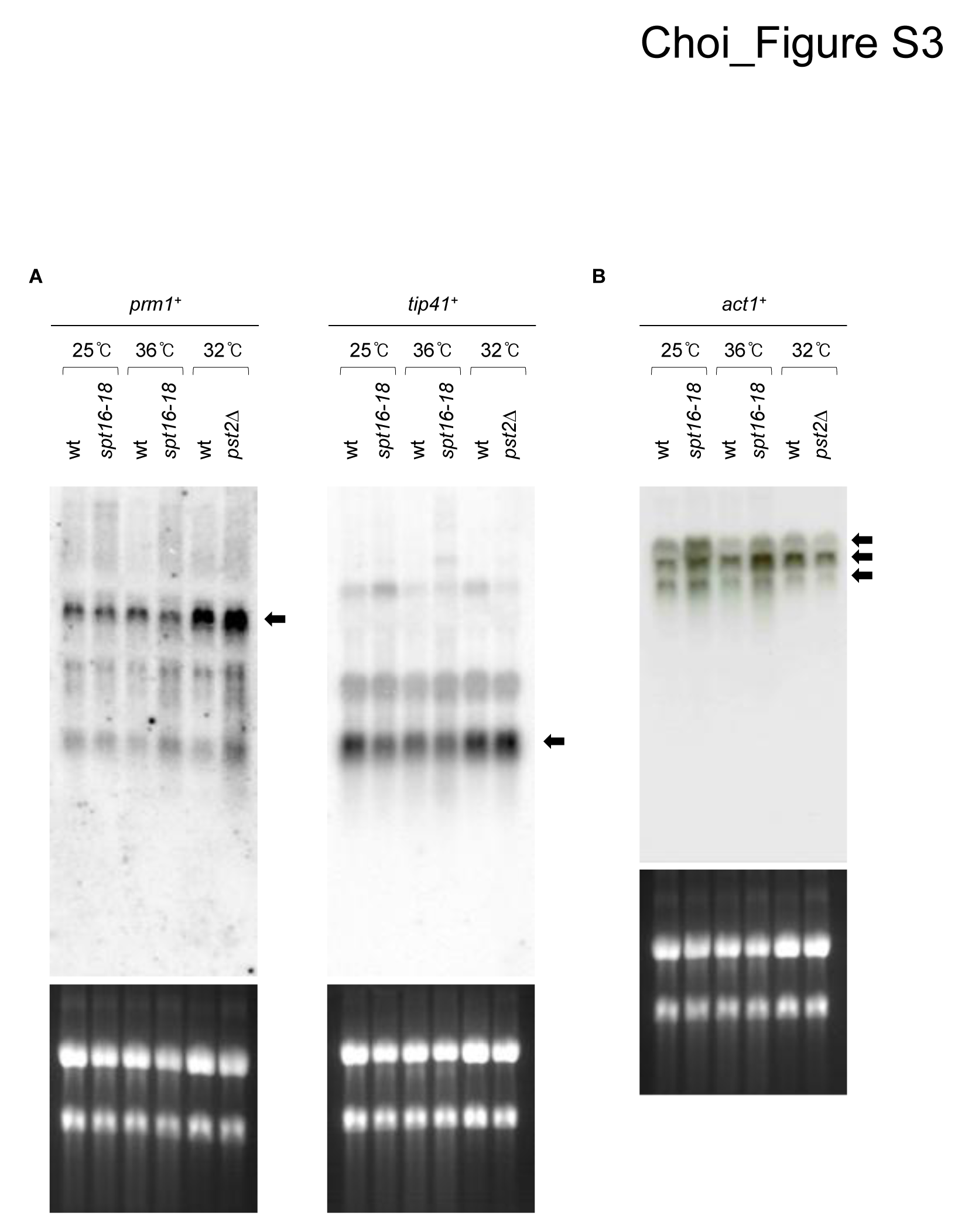

Supplement: Figure S3 — Detection of cryptic shorter transcripts from prm1 +, tip41 + and act1 +. (A) Northern analyses of transcripts from prm1 + and tip41 + gene. (B) Northern analyses of transcripts from act1 + gene. RNA was extracted from cells grown at 25°C (wt, spt16-18), 32°C for 6 h (wt, pst2Δ) or 36°C for 1 h (wt, spt16-18) after shift from 25°C. Arrow indicates full-length transcripts. (TIF) [file pgen.1002985.s003.tif]

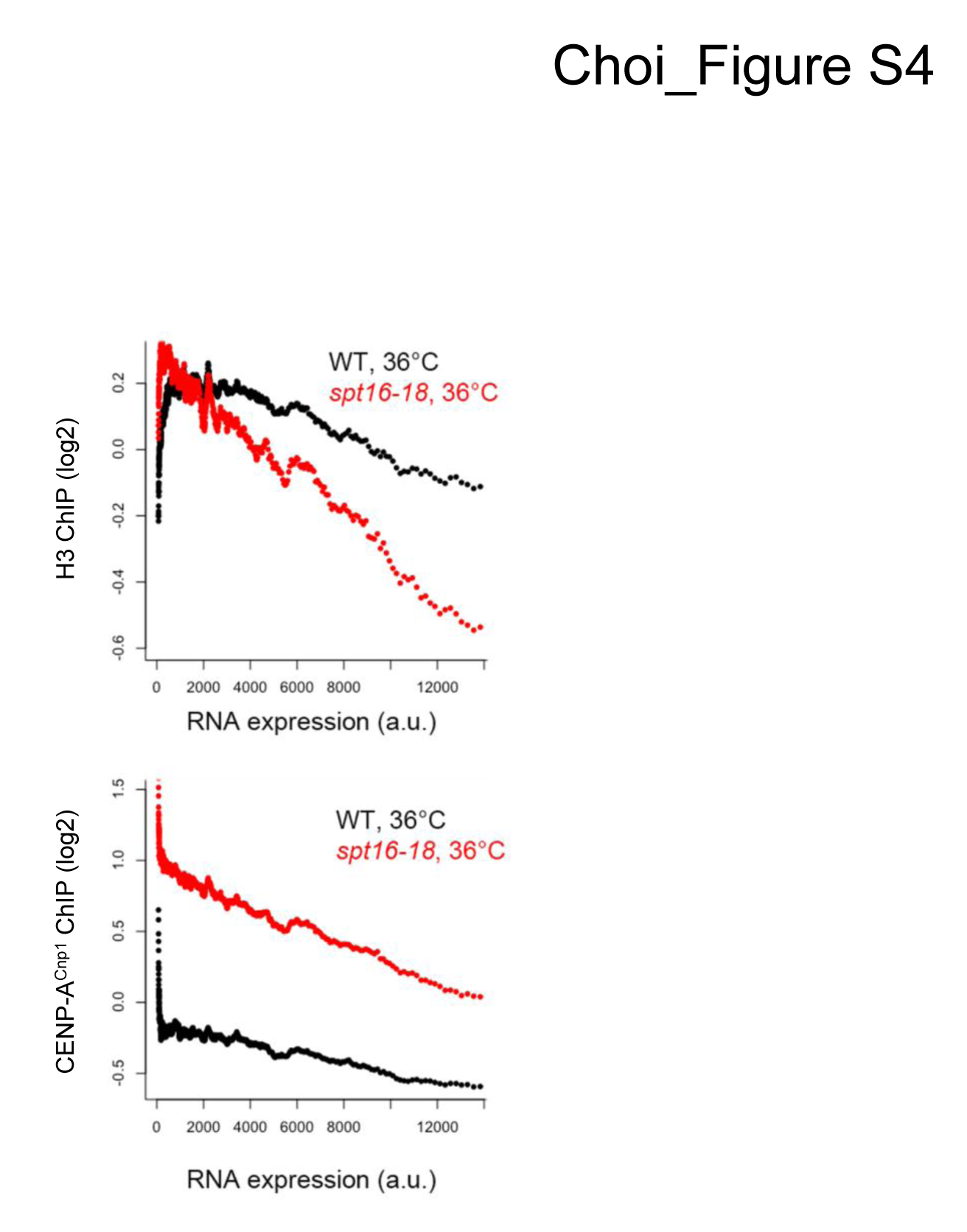

Supplement: Figure S4 — H3 and CENP-ACnp1 are preferentially incorporated in genes expressed at low to intermediate levels in spt16-18 cells. Moving average plots (window size = 100, step size = 1) of H3 (upper panel) and CENP-ACnp1 (lower panel) plotted as a function of RNA expression in WT (arbitrary units a.u.). H3/CENP-ACnp1 association in spt16-18 cells (red) and WT (black) at 36°C. (TIF) [file pgen.1002985.s004.tif]

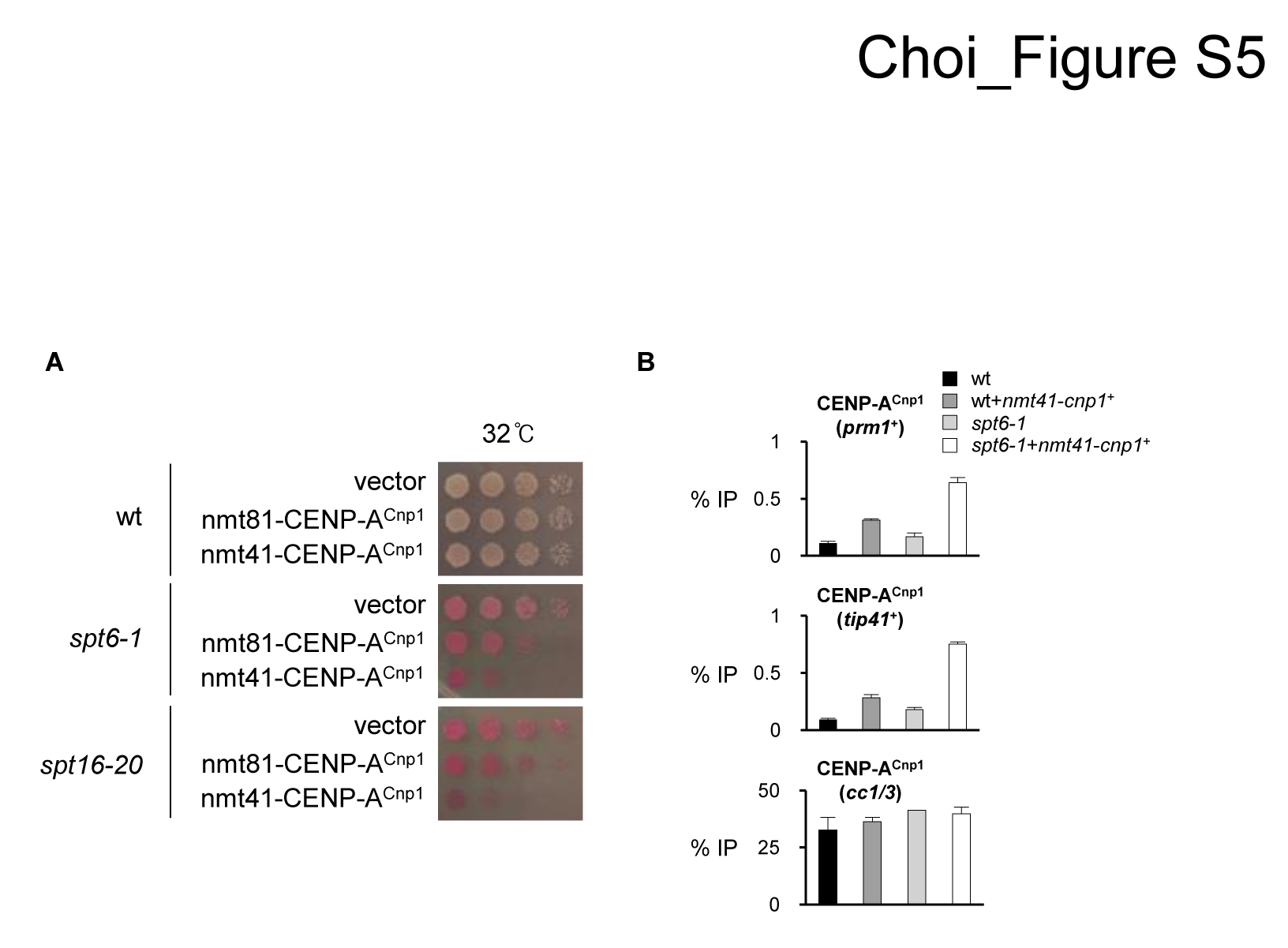

Supplement: Figure S5 — Effects of CENP-ACnp1 overexpression in cells with defective Spt6. (A) Viability of wt, spt6-1 and spt16-20 cells expressing additional CENP-ACnp1 at low (nmt81-CENP-ACnp1) and medium (nmt41-CENP-ACnp1) levels compared to empty vector at 32°C. Note: spt16-20 cells have a semi-permissive temperature similar to that of spt6-1 and thus are used as a positive control in this experiment. (B) ChIP analysis of CENP-ACnp1 levels at prm1 +, tip41 + and endogenous centromeres (cc1/3) in wt and spt6-1 cells in the absence or presence of OE-CENP-ACnp1 (nmt41-cnp1 +). Cells were grown at 36°C for 1 h after shift from 25°C. (TIF) [file pgen.1002985.s005.tif]

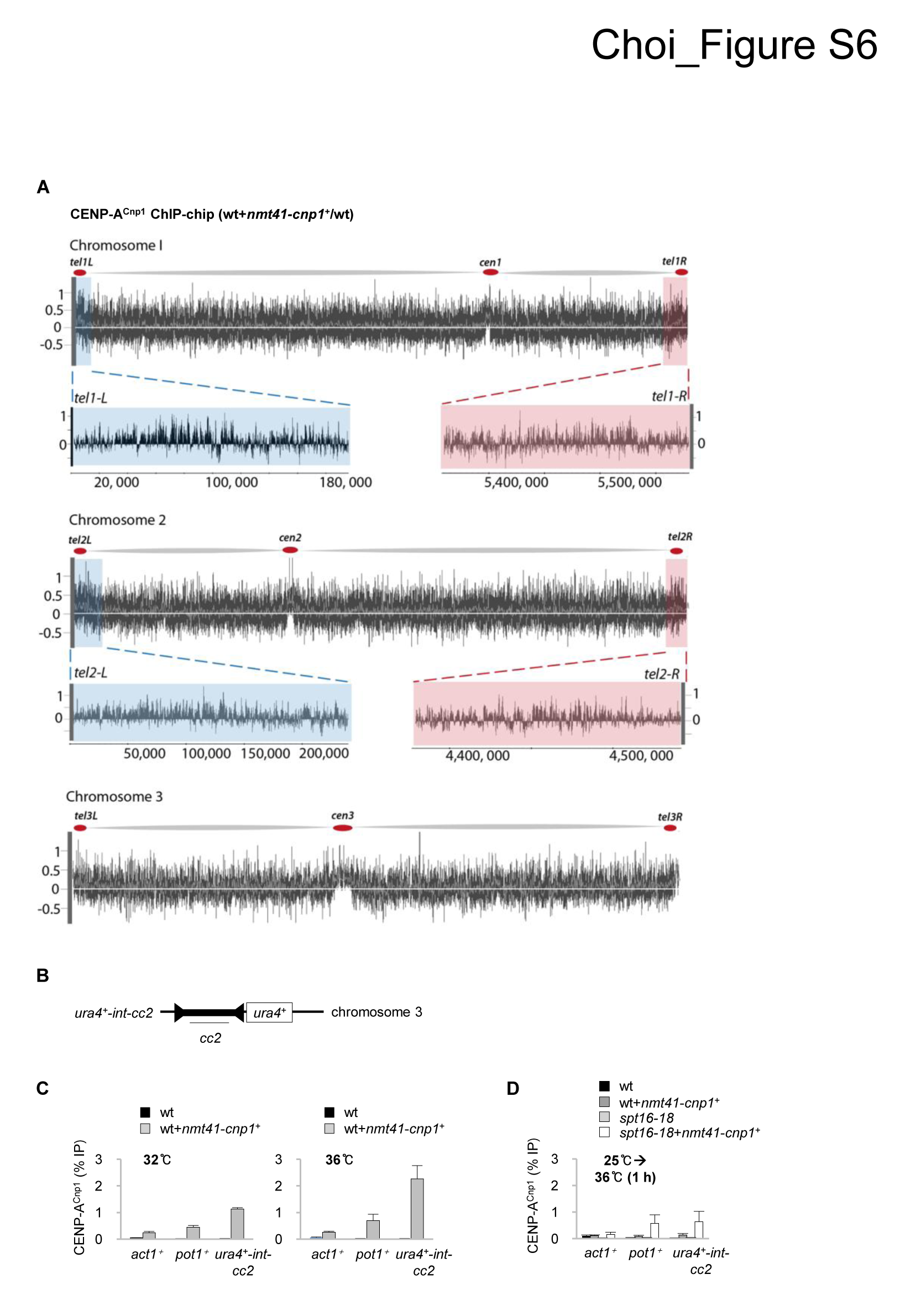

Supplement: Figure S6 — CENP-ACnp1 preferentially accumulates at subtelomeric regions and ectopically placed central domain DNA when overexpressed. (A) ChIP-chip analyses of relative levels of CENP-ACnp1 in wt cells with OE-CENP-ACnp1 (nmt41-cnp1 +) compared to wt cells without OE-CENP-ACnp1. Cells were grown at 36°C for 1 h after shift from 25°C. Data on the Y-axis are presented in log2 scale. (B) Schematic of ectopic cc2 inserted at ura4 + locus (ura4 + -int-cc2). (C) ChIP analysis of CENP-ACnp1 levels at act1 +, pot1 + and ura4 + -int-cc2 in wt cells in the absence or presence of OE-CENP-ACnp1 (nmt41-cnp1 +) grown at indicated temperatures. (D) ChIP analysis of CENP-ACnp1 levels at act1 +, pot1 + and ura4 + -int-cc2 in wt and spt16-18 cells in the absence or presence of OE-CENP-ACnp1 (nmt41-cnp1 +). Cells were grown at 36°C for 1 h after shift from 25°C. (TIF) [file pgen.1002985.s006.tif]

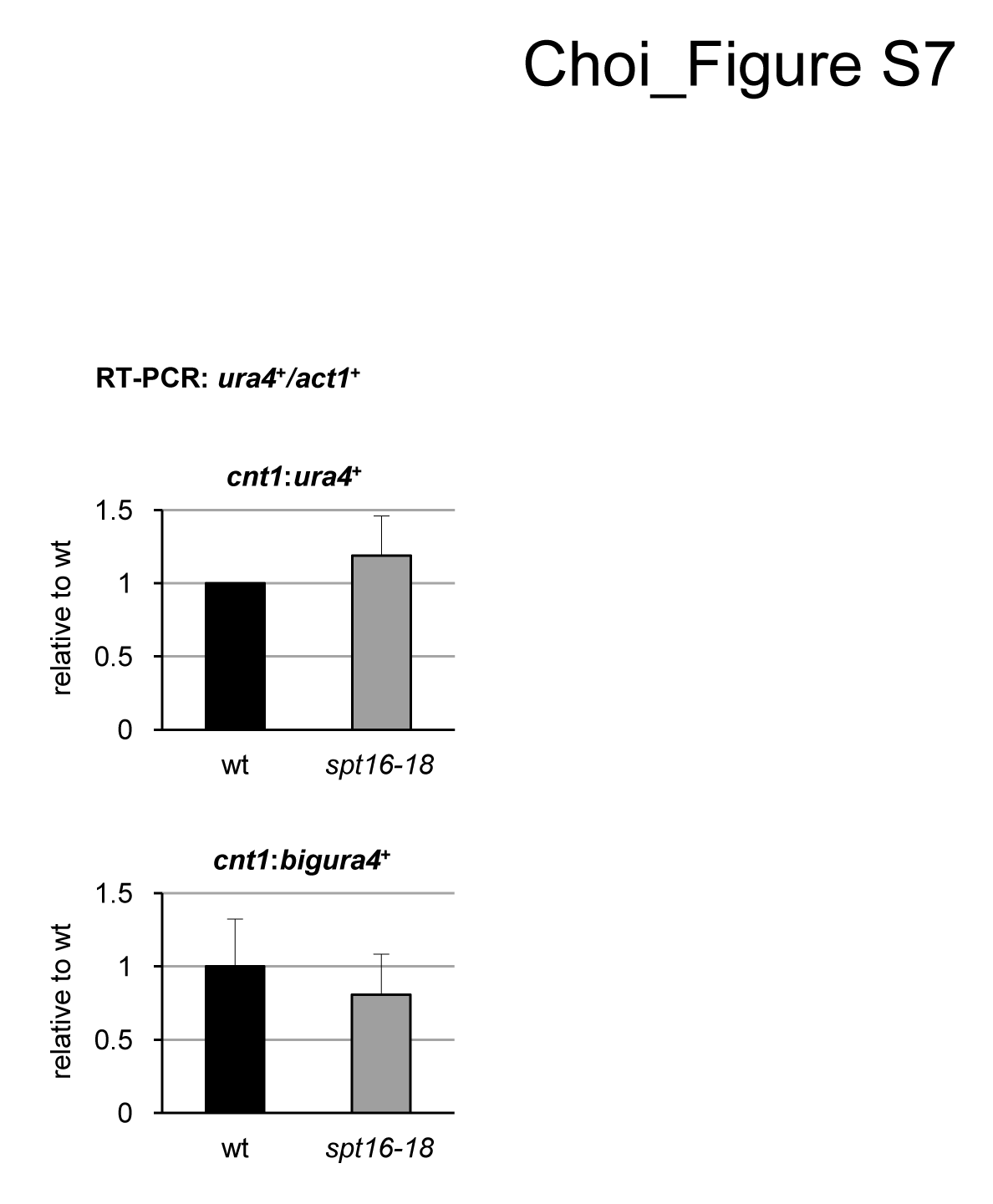

Supplement: Figure S7 — Expression of ura4 + from cnt1:ura4 + or cnt1:bigura4 + is not significantly affected in spt16-18 cells. qRT-PCR analyses to measure the levels of ura4 + transcripts from cnt1:ura4 + or cnt1:bigura4 + in wild-type and spt16-18 cells. Cells were grown at 36°C for 1 h after shift from 25°C. The relative expression levels were calculated as the value of ura4 + expression relative to act1 +. These values (ura4 +/act1 +) were further normalized to those of respective wild-type (relative to wt). Error bars indicate S.D. from 2 biological replicates. (TIF) [file pgen.1002985.s007.tif]

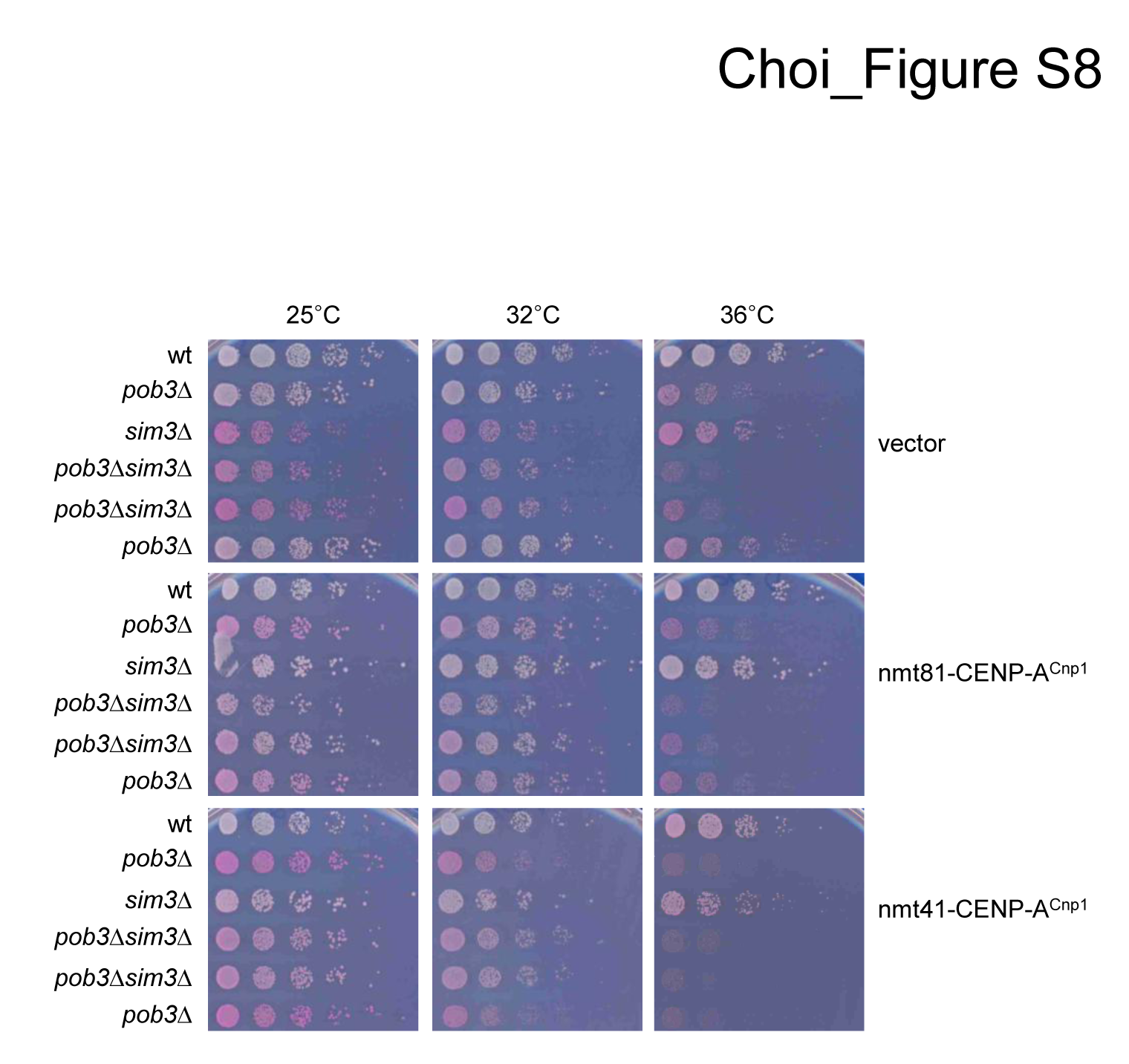

Supplement: Figure S8 — Loss of Sim3 relieves the toxic effects of CENP-ACnp1 overexpression in pob3Δ cells. Viability of wt, pob3Δ, sim3Δ and pob3Δ sim3Δ cells expressing additional CENP-ACnp1 at low (nmt81-CENP-ACnp1) or medium (nmt41-CENP-ACnp1) levels compared to empty vector. Cells were grown at 25°C, 32°C or 36°C. Phloxine B plates stain dead cells red. (TIF) [file pgen.1002985.s008.tif]

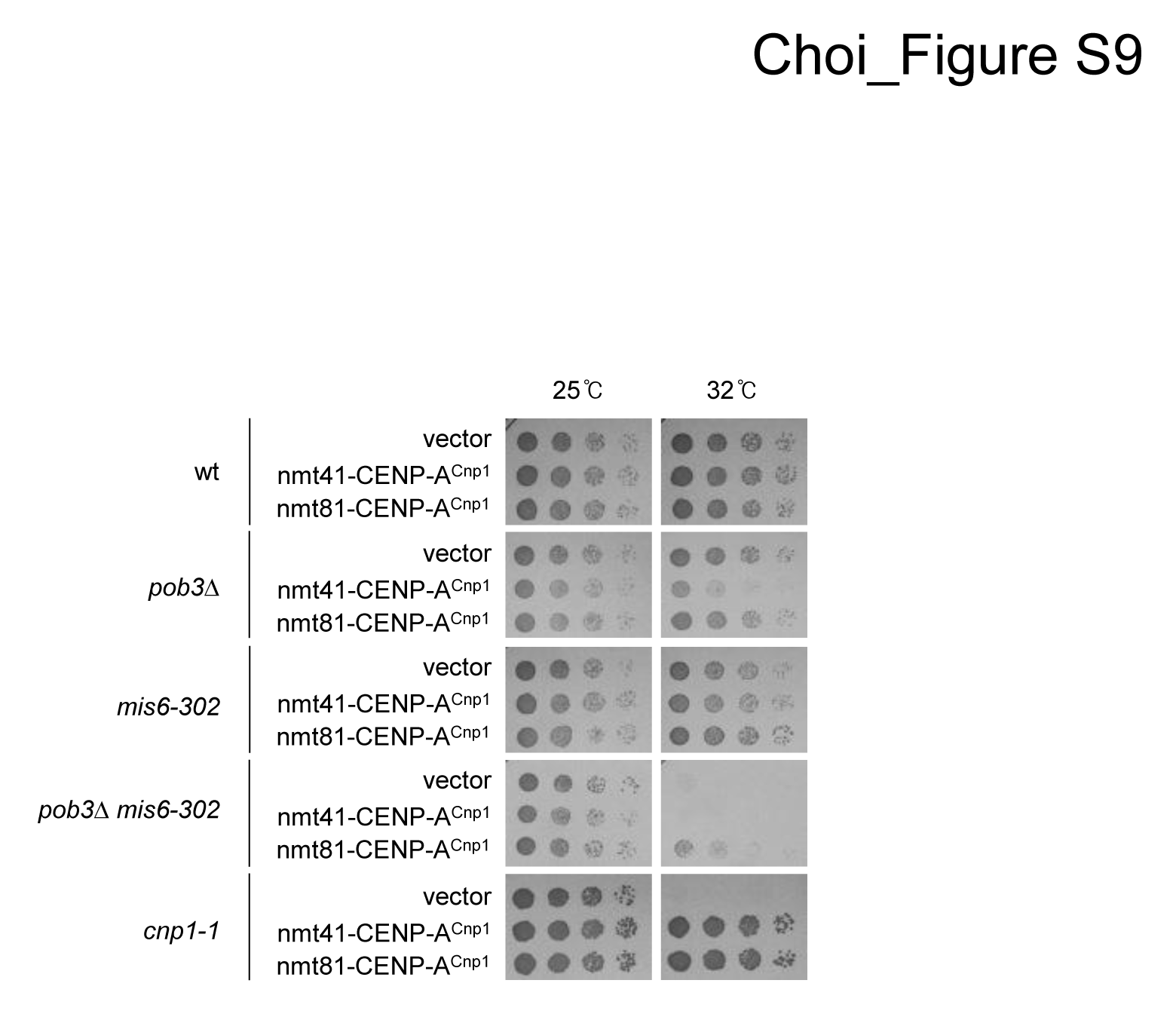

Supplement: Figure S9 — Low level overexpression of CENP-ACnp1 partially rescues the lethality of pob3Δ mis6-302 cells. Viability of wt, pob3Δ, mis6-302, pob3Δ mis6-302 and cnp1-1 strains expressing additional CENP-ACnp1 at medium (nmt41-CENP-ACnp1) or low (nmt81-CENP-ACnp1) levels compared to empty vector at 25°C or 32°C. (TIF) [file pgen.1002985.s009.tif]

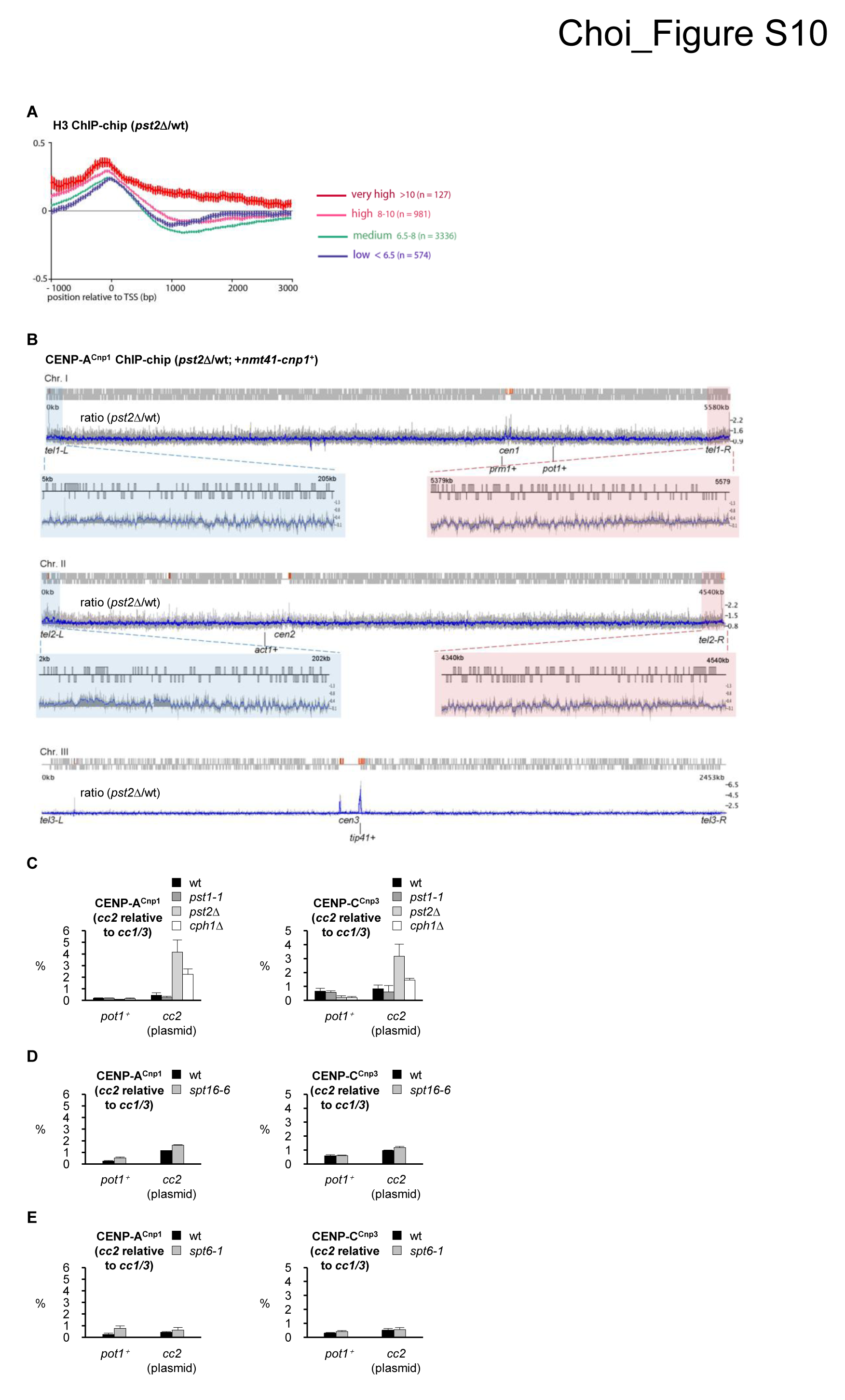

Supplement: Figure S10 — Defective function of Clr6-CII allows assembly of CENP-ACnp1 chromatin at specific locations. (A) Average gene analysis for the ratio of H3 occupancy in pst2Δ mutants versus wt. Genes are aligned at transcription start site and divided into four groups dependent of their transcription levels. n = number of genes in each group. Error bars represent 99% confidence intervals. (B) ChIP-chip analyses of relative CENP-ACnp1 levels in pst2Δ cells compared to wt in the presence of OE-CENP-ACnp1 (nmt41-cnp1 +). ORFs are displayed as grey boxes. Regions of at least 1 kb in length and with >2-fold increase in CENP-ACnp1 signal above genome-wide average are colored red. Data on the Y-axis are presented in linear scale. Blue: running average signal/100 probes. Grey: signal for individual probes. (C) ChIP analyses of CENP-ACnp1 and CENP-CCnp3 levels at pot1 + and cc2 in pcc2 plasmid compared to endogenous centromere (cc1/3) in wt, pst2Δ, cph1Δ and pst1-1 cells carrying pcc2. Cells were collected after 30 cell doublings at 32°C from the introduction of pcc2. Error bar indicates standard deviation from 3-4 independent biological experiments. (D) ChIP analyses of CENP-ACnp1 and CENP-CCnp3 levels at pot1 + and cc2 in pcc2 plasmid compared to endogenous centromere (cc1/3) in wt and spt16-6 cells carrying pcc2. Error bar indicates standard deviation from 3 independent biological experiments. (Note: we find that most of spt16-ts alleles including spt16-18 do not allow efficient propagation of pcc2 plasmid and thus a specific allele (spt16-6) which allows propagation of pcc2 is used in this particular assay.) (E) ChIP analyses of CENP-ACnp1 and CENP-CCnp3 levels at pot1 + and cc2 in pcc2 plasmid compared to endogenous centromere (cc1/3) in wt and spt6-1 cells carrying pcc2. Error bar indicates standard deviation from 3 independent biological experiments. (TIF) [file pgen.1002985.s010.tif]

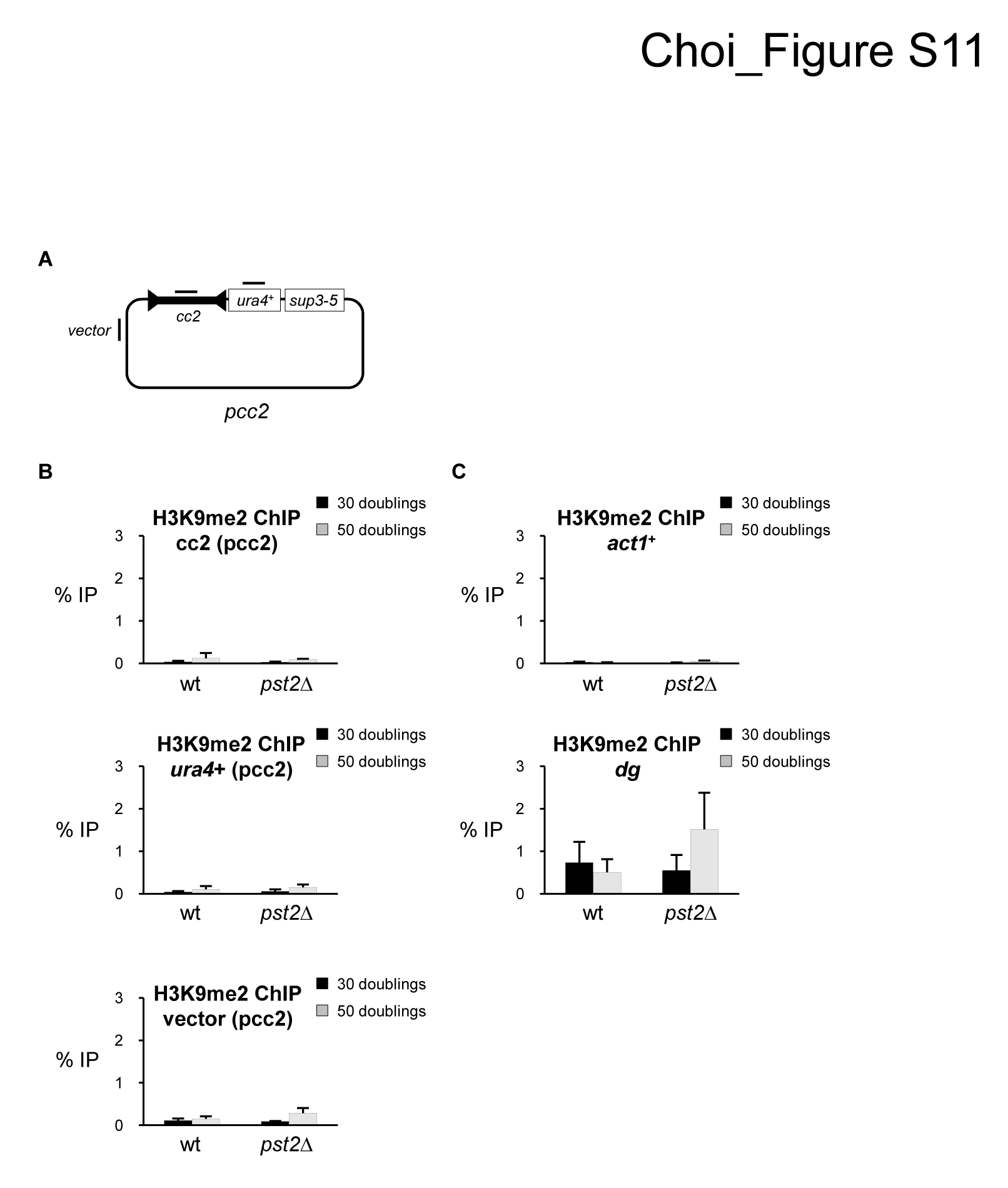

Supplement: Figure S11 — Loss of Clr6-CII function does not induce H3K9 methylation on pcc2 plasmid. (A) Schematic of pcc2 plasmid. Regions amplified by primer pairs used in ChIP-qPCR (cc2, ura4 + and vector - a region on the plasmid backbone) are indicated as short black bars. (B) ChIP analyses of H3K9 methylation (H3K9me2) levels at cc2, ura4 + and vector in pcc2 in wt and pst2Δ cells carrying pcc2. (C) ChIP analyses of H3K9me2 levels at chromosomal act1 + and dg in the same samples. dg represents a part of heterochromatic centromere outer repeats and thus serves as a positive control for H3K9me2 ChIP. ChIP was performed after 30 and 50 cell doublings at 32°C from the introduction of pcc2. Enrichment is reported as % IP. Error bars indicate S.D. from 4 biological replicates. (TIF) [file pgen.1002985.s011.tif]
