# Supplementary material for: Factors That Promote H3 Chromatin Integrity during Transcription Prevent Promiscuous Deposition of CENP-ACnp1 in Fission Yeast
Source: PLoS Genet. 2012 Sep 20;8(9):e1002985. doi: 10.1371/journal.pgen.1002985 (PMC3447972; doi:10.1371/journal.pgen.1002985)
Supplement: Table S1 — Relative enrichment of CENP-ACnp1 and H3 in spt16-18 versus wild-type cells (at 36°C) at selected genes from ChIP-chip data and their relative RNA expression levels (at 30°C; transcription levels were categorized as in Figure 2F). (DOC) [file pgen.1002985.s012.doc]

**Table S1. Relative enrichment of CENP-A^Cnp1^ and H3 in *spt16-18* versus wild-type cells (at 36°C) at selected genes from ChIP-chip data and their relative RNA expression levels (at 30°C; transcription levels were categorized as in Figure 2F)**

| genes | CENP-A^Cnp1^  (*spt16-18*/wt) | H3  (*spt16-18*/wt) | Transcription level |
| --- | --- | --- | --- |
| *act1^+^* | 0.2 | -1.09 | very high |
| *pot1^+^* | 1.07 | 0.11 | medium |
| *prm1^+^* | 1.75 | 0 | high |
| *tip41^+^* | 2.06 | 0.26 | very low |
| *SPBC19C7.11* | 1.01 | 0.12 | high |
| *msh1^+^* | 1 | -0.04 | medium |

All values are in log2.

(Note: Only CENP-A^Cnp1^ data is absolute, i.e. made with spiked in controls.)
