# Supplementary material for: Factors That Promote H3 Chromatin Integrity during Transcription Prevent Promiscuous Deposition of CENP-ACnp1 in Fission Yeast
Source: PLoS Genet. 2012 Sep 20;8(9):e1002985. doi: 10.1371/journal.pgen.1002985 (PMC3447972; doi:10.1371/journal.pgen.1002985)
Supplement: Table S2 — List of strains. (DOC) [file pgen.1002985.s013.doc]

**Table S2. List of Strains**

(*Figure indicates the experiment in which the strain is used for the first time)

| Figure* | Strain | Genotype (only the relevant genotype is listed) |
| --- | --- | --- |
| Fig. S1B | 336 | *cnt1:ura4^+^ ura4-DS/E* |
| Fig. S1B | 4415 | *cnt1:ura4^+^ cnp1-87 ura4-DS/E* |
| Fig. S1B | A3026 | *cnt1:ura4^+^ spt16-1-kan^R^ ura4-DS/E* |
| Fig. S1B | A3028 | *cnt1:ura4^+^ spt16-2-kan^R^ ura4-DS/E* |
| Fig. S1B | A3030 | *cnt1:ura4^+^ spt16-3-kan^R^ ura4-DS/E* |
| Fig. S1B | A3032 | *cnt1:ura4^+^ spt16-4-kan^R^ ura4-DS/E* |
| Fig. S1B | A3034 | *cnt1:ura4^+^ spt16-5-kan^R^ ura4-DS/E* |
| Fig. S1B | A3036 | *cnt1:ura4^+^ spt16-6-kan^R^ ura4-DS/E* |
| Fig. S1B | A3149 | *cnt1:ura4^+^ spt16-7-kan^R^ ura4-DS/E* |
| Fig. S1B | A3038 | *cnt1:ura4^+^ spt16-8-kan^R^ ura4-DS/E* |
| Fig. S1B | A3040 | *cnt1:ura4^+^ spt16-9-kan^R^ ura4-DS/E* |
| Fig. S1B | A3042 | *cnt1:ura4^+^ spt16-10-kan^R^ ura4-DS/E* |
| Fig. S1B | A3151 | *cnt1:ura4^+^ spt16-11-kan^R^ ura4-DS/E* |
| Fig. S1B | A3153 | *cnt1:ura4^+^ spt16-12-kan^R^ ura4-DS/E* |
| Fig. S1B | A3155 | *cnt1:ura4^+^ spt16-13-kan^R^ ura4-DS/E* |
| Fig. S1B | A3157 | *cnt1:ura4^+^ spt16-14-kan^R^ ura4-DS/E* |
| Fig. S1B | A3044 | *cnt1:ura4^+^ spt16-15-kan^R^ ura4-DS/E* |
| Fig. S1B | A3159 | *cnt1:ura4^+^ spt16-17-kan^R^ ura4-DS/E* |
| Fig. S1B | A3162 | *cnt1:ura4^+^ spt16-18-kan^R^ ura4-DS/E* |
| Fig. S1B | A3163 | *cnt1:ura4^+^ spt16-19-kan^R^ ura4-DS/E* |
| Fig. S1B | A3165 | *cnt1:ura4^+^ spt16-20-kan^R^ ura4-DS/E* |
| Fig. S1B | A3167 | *cnt1:ura4^+^ spt16-21-kan^R^ ura4-DS/E* |
| Fig. S1B | A3169 | *cnt1:ura4^+^ spt16-22-kan^R^ ura4-DS/E* |
| Fig. S1B | A3171 | *cnt1:ura4^+^ spt16-23-kan^R^ ura4-DS/E* |
| Fig. S1B | A3046 | *cnt1:ura4^+^ spt16-24-kan^R^ ura4-DS/E* |
| Fig. S1C | A841 | *cnt1:ura4^+^ pob3*Δ::*nat^R^ ura4-DS/E* |
| Fig. S1D | 972 | wild-type *S. pombe* |
| Fig. S1D | 9144 | *pob3*Δ::*nat^R^* |
| Fig. S1D | 4761 | *cnp1-87* |
| Fig. S1D | A1410 | *pob3*Δ::*nat^R^ cnp1-87* |
| Fig. 1A | 1645 | *ade6-210 ura4-D18 leu1-32 arg3-D3 his3-D1* |
| Fig. 1C | 5205 | *ars1(Mlu*I*):41x-GFP-cnp1^+^-LEU2 leu1-32* |
| Fig. 1C | A5045 | *ars1(Mlu*I*):41x-GFP-cnp1^+^-LEU2 leu1-32 spt16-18-kan^R^* |
| Fig. 1C | KT1769 | *cnp1^+^::GFP-cnp1^+^-nat^R^* **(1)** |
| Fig. 1C | A8155 | *cnp1^+^::GFP-cnp1^+^-nat^R^ spt16-18-kan^R^* |
| Fig. 1C | A7383 | *ars1(Mlu*I*):81x-GFP-cnp1^+^-nat^R^* |
| Fig. 1C | A8157 | *ars1(Mlu*I*):81x-GFP-cnp1^+^-nat^R^ spt16-18-kan^R^* |
| Fig. 2B | A5259 | *spt16-18-kan^R^ leu1-32* |
| Fig. 2B | A2858 | *pst2*Δ::*kan^R^* |
| Fig. 2C | A6187 | *cph1*Δ::*nat^R^* |
| Fig. 2C | A6184 | *alp13*Δ::*kan^R^* |
| Fig. 2C | 2508 | *pst1-1* |
| Fig. 2D | A1243 | *htb1-FLAG-kan^R^* **(2)** |
| Fig. 2D | A7514 | *htb1-FLAG-kan^R^ spt16-18-kan^R^* |
| Fig. 3A | A326 | *ars1(Mlu*I*):3x-LEU2 leu1-32* |
| Fig. 3A | A334 | *ars1(Mlu*I*):41x-cnp1^+^-LEU2 leu1-32* |
| Fig. 3A | A6869 | *ars1(Mlu*I*):3x-LEU2 leu1-32 spt16-18-kan^R^* |
| Fig. 3A | A6871 | *ars1(Mlu*I*):41x-cnp1^+^-LEU2 leu1-32 spt16-18-kan^R^* |
| Fig. 3G | A7238 | *clr4*Δ::*nat^R^ ars1(Mlu*I*):41x-cnp1^+^-LEU2 leu1-32* |
| Fig. 3G | A7240 | *clr4*Δ::*nat^R^ spt16-18-kan^R^ ars1(Mlu*I*):41x-cnp1^+^-LEU2 leu1-32* |
| Fig. 3G | A7242 | *cen1*Δ::*kan^R^ tel1R-neocen ars1(Mlu*I*):41x-cnp1^+^-LEU2 leu1-32* **(3)** |
| Fig. 3G | A7244 | *cen1*Δ::*kan^R^ spt16-18-kan^R^ tel1R-neocen ars1(Mlu*I*):41x-cnp1^+^-LEU2 leu1-32* |
| Fig. S5A | A6793 | *spt6-1-nat^R^* **(4)** |
| Fig. S5B | A6873 | *spt6-1-nat^R^ ars1(Mlu*I*):3x-LEU2 leu1-32* |
| Fig. S5B | A6875 | *spt6-1-nat^R^ ars1(Mlu*I*):41x-cnp1^+^-LEU2 leu1-32* |
| Fig. S5C | A7169 | *hht2*Δ*::ura4^+^ hht3*Δ*::kan^R^ ura4-D18 or DS/E* |
| Fig. S6C | A6909 | *ura4*Δ*::nat^R^-cc2-ura4^+^ ars1(Mlu*I*):3x-LEU2 leu1-32 cc2:his3^+^* |
| Fig. S6C | A7153 | *ura4*Δ*::nat^R^-cc2-ura4^+^ ars1(Mlu*I*):41x-cnp1^+^-LEU2 leu1-32 cc2:his3^+^* |
| Fig. S6D | A6951 | *ura4*Δ*::nat^R^-cc2-ura4^+^ spt16-18-kan^R^ ars1(Mlu*I*):3x-LEU2 leu1-32 cc2:his3^+^* |
| Fig. S6D | A7157 | *ura4*Δ*::nat^R^-cc2-ura4^+^ spt16-18-kan^R^ ars1(Mlu*I*):41x-cnp1^+^-LEU2 leu1-32 cc2:his3^+^* |
| Fig. 4B | 4638 | *cnt1:bigura4^+^ ura4-DS/E* |
| Fig. 4B | A6557 | *cnt1:bigura4^+^ spt16-17-kan^R^ ura4-DS/E* |
| Fig. 4B | A6560 | *cnt1:bigura4^+^ spt16-18-kan^R^ ura4-DS/E* |
| Fig. 5A | A1479 | *scm3-15* |
| Fig. 5A | A7320 | *pob3*Δ::*nat^R^ scm3-15* |
| Fig. 5A | 2919 | *mis6-302* **(5)** |
| Fig. 5A | A853 | *pob3*Δ::*nat^R^ mis6-302* |
| Fig. 5A | A592 | *mis18-262* **(5)** |
| Fig. 5A | A7258 | *pob3*Δ::*nat^R^ mis18-262* |
| Fig. 5A | 3828 | *mis12-537* **(5)** |
| Fig. 5A | A871 | *pob3*Δ::*nat^R^ mis12-537* |
| Fig. 5B | A7323 | *pob3*Δ::*nat^R^ clr4*Δ::*LEU2* |
| Fig. 5B | A7405 | *clr4*Δ::*LEU2 mis6-302* |
| Fig. 5B | A7324 | *pob3*Δ::*nat^R^ clr4*Δ::*LEU2 mis6-302* |
| Fig. 5E | 6960 | *lys1^+^-cnp1-1 cnp1*Δ*::ura4^+^* **(5)** |
| Fig. S8 | A1047 | *sim3*Δ::*kan^R^* |
| Fig. S8 | A8242 | *sim3*Δ::*kan^R^ pob3*Δ::*nat^R^* |
| Fig. 6A | A6553 | *h^-^ spt16-17-kan^R^* |
| Fig. 6C | A6865 | *pst2*Δ::*kan^R^ ars1(Mlu*I*):3x-LEU2 leu1-32* |
| Fig. 6C | A6867 | *pst2*Δ::*kan^R^ ars1(Mlu*I*):41x-cnp1^+^-LEU2 leu1-32* |
| Fig. 6E | A2213 | *cc2:his3^+^ ade6-704-hyg^R^* |
| Fig. 6E | A6479 | *pst2*Δ::*kan^R^ cc2:his3^+^ ade6-704-hyg^R^* |
| Fig. S10C | A6481 | *cph1*Δ::*nat^R^ cc2:his3^+^ ade6-704-hyg^R^* |
| Fig. S10C | A6370 | *pst1-1 cc2:his3^+^ ade6-704-hyg^R^* |
| Fig. S10D | A5603 | *spt16-6-kan^R^ cc2:his3^+^ ade6-704-hyg^R^* |
| Fig. S10E | A6824 | *spt6-1-nat^R^ cc2:his3^+^ ade6-704-hyg^R^* |

Original source:

(1) Kohta Takahashi

(2) Shiv Grewal

(3) Kojiro Ishii (original strain = *cen1*Δ::*kan^R^ cd60 tel1R-neocen*)

(4) Fred Winston

(5) Mitsuhiro Yanagida
