# Supplementary material for: Factors That Promote H3 Chromatin Integrity during Transcription Prevent Promiscuous Deposition of CENP-ACnp1 in Fission Yeast
Source: PLoS Genet. 2012 Sep 20;8(9):e1002985. doi: 10.1371/journal.pgen.1002985 (PMC3447972; doi:10.1371/journal.pgen.1002985)
Supplement: Table S3 — List of primers. (DOC) [file pgen.1002985.s014.doc]

**Table S3. List of Primers**

(*Figure indicates the figure in which the primer is used for the first time)

| Figure* | Primer | Sequence |
| --- | --- | --- |
| Fig. 2B | SPBC19C7.11-for | attggcaatatgctgggaaa |
| Fig. 2B | T7-SPBC19C7.11-rev | TAATACGACTCACTATAGGGAGAcgccaactgaatcaaccttt |
| Fig. 2B | pot1-for | gtttggatgtttgccttgct |
| Fig. 2B | T7-pot1-rev | TAATACGACTCACTATAGGGAGAcaattttcgtgccaaatcct |
| Fig. 2B | msh1-for | cgcaaattggaagctttgtt |
| Fig. 2B | T7-msh1-rev | TAATACGACTCACTATAGGGAGAttgagcccgtgtgattgata |
| Fig. S3A | prm1-probe-for | CAGCACAGTGAGTTTCTCTGG |
| Fig. S3A | T7-prm1-probe-rev | TAATACGACTCACTATAGGGAGATGAAATAATTGCCGGTGAGA |
| Fig. S3A | tip41-probe-for | CTGGGCCAAATCCAGAATTA |
| Fig. S3A | T7-tip41-probe-rev | TAATACGACTCACTATAGGGAGAAGCAATGCAGCTTTGTCTCC |
| Fig. S3B | act1-probe-for | cgaacgtgaaattgttcgtg |
| Fig. S3B | T7-act1-probe-rev | TAATACGACTCACTATAGGGAGAaatggatccaccaatccaga |
| Fig. 2D | qact1-for (*act1*^+^) | cccaaatccaaccgtgagaagatg |
| Fig. 2D | qact1-rev (*act1*^+^) | ccagagtccaagacgataccagtg |
| Fig. 2D | pot1-for (*pot1*^+^) | gtttggatgtttgccttgct |
| Fig. 2D | qpot1-rev (*pot1*^+^) | caagttcccccaaatcagaa |
| Fig. 3A | qCnt1-F (*cc1/3*) | CAGACAATCGCATGGTACTATC |
| Fig. 3A | qCnt1-R (*cc1/3*) | AGGTGAAGCGTAAGTGAGTG |
| Fig. 3D | qprm1-for (*prm1*^+^) | GATTCGCTGGAGAAAGTTGC |
| Fig. 3D | qprm1-rev (*prm1*^+^) | CGGAGAGACTGGATTTCAGG |
| Fig. 3D | qtip41-for (*tip41*^+^) | CACGCCTTGTCGTACGTTTA |
| Fig. 3D | qtip41-rev (*tip41*^+^) | ACGGCAGTCCTTCAAGAGAA |
| Fig. 3G | qSPAC869.03c-for (*tel1R*) | gatttggccaagcaagtcat |
| Fig. 3G | qSPAC869.03c-rev (*tel1R*) | agaagcatggcagaaacgat |
| Fig. 4B | qcnt2-f1 (*cc2*) | cattaaacaaacaacggcacac |
| Fig. 4B | qcnt2-r1 (*cc2*) | taagccagcaaattccttgag |
| Fig. 4B | qura4-f2 (*ura4*^+^) | aggctctttggctactggttcc |
| Fig. 4B | qura4-r2 (*ura4*^+^) | agttatgtagtcgctttgaaggttagg |
| Fig. S11B | pDF16-vec-f2 | ataataccgcgccacatagc |
| Fig. S11B | pDF16-vec-r2 | ccagaaacgctggtgaaagt |
| Fig. S11C | qDgI-fw (*dg*) | AATTGTGGTGGTGTGGTAATAC |
| Fig. S11C | qDgI-rev (*dg*) | GGGTTCATCGTTTCCATTCAG |
