# Supplementary material for: Factors That Promote H3 Chromatin Integrity during Transcription Prevent Promiscuous Deposition of CENP-ACnp1 in Fission Yeast
Source: PLoS Genet. 2012 Sep 20;8(9):e1002985. doi: 10.1371/journal.pgen.1002985 (PMC3447972; doi:10.1371/journal.pgen.1002985)
Supplement: Table S4 — List of spt16-ts alleles. (DOC) [file pgen.1002985.s015.doc]

**Table S4. List of *spt16-ts* alleles**

(Amino acid changes, position of kan^R^ insertion and growth defect at 32°C associated with *spt16-ts* alleles)

| Allele | Amino acid changes | kan^R^ insertion | Growth at 32°C |
| --- | --- | --- | --- |
| *spt16-1* | E74K, K308R, N375D, L394S | upstream | + |
| *spt16-2* | L173P, N293K, A315V, L387P | upstream | - |
| *spt16-3* | D201E, K264R, I362V, A381V, L385P | upstream | ++ |
| *spt16-4* | D164G, C281R, D448N | upstream | - |
| *spt16-5* | A2S, I71R, R484G | upstream | + |
| *spt16-6* | G361V | upstream | ++ |
| *spt16-7* | A187T, S311P | upstream | - |
| *spt16-8* | K176E, L409F | upstream | ++ |
| *spt16-9* | L340P | upstream | ++ |
| *spt16-10* | L348P, A358V | upstream | -- |
| *spt16-11* | L225R, S292P, P302S | upstream | + |
| *spt16-12* | D47N, M195T, P452T, F504V | upstream | + |
| *spt16-13* | Q782R, L893P | downstream | -- |
| *spt16-14* | P552L, D625G, E776D, G858S, D870Y | downstream | - |
| *spt16-15* | F813S, T835R, V855I, D897V | downstream | + |
| *spt16-16* | I878N, L893F | downstream | -- |
| *spt16-17* | F583I | downstream | + |
| *spt16-18* | V747A, D870G, I881N, T915A | downstream | -- |
| *spt16-19* | I553N | downstream | ++ |
| *spt16-20* | P559L, F609C, E773G | downstream | - |
| *spt16-21* | V558D, K630M | downstream | + |
| *spt16-22* | Y690C,H724R | downstream | + |
| *spt16-23* | A812V, P838L, I881T | downstream | -- |
| *spt16-24* | Q742H, D836N, I878S, E925D | downstream | ++ |
| *spt16-25* | Y544C, R556G | downstream | ++ |
